# Comparative Analysis of the Pattern of Population Genetic Diversity in Three Indo-West Pacific *Rhizophora* Mangrove Species

**DOI:** 10.3389/fpls.2016.01434

**Published:** 2016-09-30

**Authors:** Yu-Bin Yan, Norm C. Duke, Mei Sun

**Affiliations:** ^1^School of Biological Sciences, The University of Hong KongHong Kong, China; ^2^The Centre for Tropical Water and Aquatic Ecosystem Research, James Cook UniversityTownsville, QLD, Australia

**Keywords:** genetic diversity, genetic drift, gene flow, inbreeding coefficient, mangroves, microsatellites, population structure, *Rhizophora*

## Abstract

*Rhizophora* species are the most widely distributed mangrove trees in the Indo-West Pacific (IWP) region. Comparative studies of these species with shared life history traits can help identify evolutionary factors that have played most important roles in determining genetic diversity within and between populations in ocean-current dispersed mangrove tree species. We sampled 935 individuals from 54 natural populations for genotyping with 13 microsatellite markers to investigate the level of genetic variation, population structure, and gene flow on a broad geographic scale in *Rhizophora apiculata, Rhizophora mucronata*, and *Rhizophora stylosa* across the IWP region. In contrast to the pattern expected of long-lived woody plants with predominant wind-pollination, water-dispersed seeds and wide geographic range, genetic variation within populations was generally low in all the three species, especially in those peripheral populations from geographic range limits. Although the large water-buoyant propagules of *Rhizophora* have capacity for long distance dispersal, such events might be rare in reality, as reflected by the low level of gene flow and high genetic differentiation between most of population pairs within each species. Phylogeographic separation of Australian and Pacific island populations from SE Asian lineages previously revealed with DNA sequence data was still detectable in *R. apiculata* based on genetic distances, but this pattern of disjunction was not always evident in *R. mucronata* and *R. stylosa*, suggesting that fast-evolving molecular markers could be more suitable for detecting contemporary genetic structure but not deep evolutionary divergence caused by historical vicariance. Given that mangrove species generally have small effective population sizes, we conclude that genetic drift coupled with limited gene flow have played a dominant role in producing the current pattern of population genetic diversity in the IWP *Rhizophora* species, overshadowing the effects of their life history traits. Recent population fragmentation and disturbances arising from human activities could further endanger genetic diversity in mangrove trees.

## Introduction

Mangroves consist of a diverse group of species of trees, shrubs, palms, vines, and ferns from 20 families (Tomlinson, 1986; Duke et al., 1998). These species can be found in 128 countries or territories, but occur most commonly in tropical and subtropical coastlines, covering an estimated total area of 152,000 square kilometers globally (Spalding et al., 2010; Duke, 2013, 2014). Mangrove forests are among the most productive ecosystems in the world with important ecological and economic values. A large number of marine and terrestrial animal species use mangroves as habitats. Various mangrove species have been used as charcoal, firewood, timber, tannins, food, or medicines by local people (FAO, 2007; UNEP, 2014). The market value of capture fisheries supported by mangroves was estimated to be 953 to 21,272 US dollars per hectare per year (Ronnback, 1999). Much of the vast biodiversity of the mangrove ecosystem depends on a few key tree species that shape the system, such as species of *Rhizophora*, the most representative genus in the mangrove family Rhizophoraceae with broad distribution in both Indo-West Pacific (IWP) and Atlantic-East Pacific (AEP) regions.

In the IWP region, only three species of *Rhizophora*—*R. apiculata* Blume, *R. mucronata* Lam., and *R. stylosa* Griff. occur naturally with wide and overlapping distributions (Duke et al., 2002). These species are primarily wind-pollinated as suggested by a large pollen/ovule ratio, short pollen presentation time and a brief functional life span of the flowers (Tomlinson et al., 1979; Nadia and Machado, 2014). Their large propagules can float in seawater for many months (Rabinowitz, 1978); hence they are perceived to have capacity for long distance dispersal by ocean currents. However, distributional and genetic discontinuities have been found at different geographic scales, both within and between IWP and AEP regions (Duke et al., 2002; Lo et al., 2014), suggesting that populations of *Rhizophora* could be highly structured due to various barriers to gene flow, such as land masses, directions of ocean currents, as well as historical vicariant events.

Although the ecological and economic values of mangroves have been increasingly appreciated, they are being destroyed at an alarming rate, 3–5 times greater than the average rates of deforestation (Duke et al., 2007; UNEP, 2014). Conservation and sustainable management of existing mangrove populations of core species are urgently needed now than ever. Genetic information is required to inform conservation practices (Frankham et al., 2002). For example, levels of genetic variation and patterns of population genetic structure are important factors influencing mangroves' long-term potential for survival, especially in the face of extensive human mediated disturbance. Analysis of inter-population gene flow and genetic distance within and between closely related taxa can be used to define species or evolutionarily significant units within species (Woodruff, 2001).

Spatial and temporal organizations of genetic variation among individuals both within and between populations are influenced by historical, demographical, ecological, and evolutionary factors as well as specific life history traits (Barrett and Shore, 1989; Hamrick and Godt, 1989, 1996; Nybom and Bartish, 2000; Rogstad and Pelikan, 2011). When the distribution of a species is not homogenous over a large geographic area, evolutionary forces, e.g., mutation, selection, and genetic drift, may act independently in each population, and genetic differentiation is subsequently developed among populations (Wakeley, 2000). Gene flow, on the other hand, is an opposing evolutionary force to genetic differentiation as it tends to homogenize genetic composition of populations and thus also plays an important role in shaping a species' genetic structure, as the extent of gene flow has a major influence on the pattern of genetic diversity both within and between populations (Slatkin, 1987; Excoffier et al., 2009; Ellstrand, 2014). Compared to terrestrial plant species, there are additional historical and environmental factors that contribute to distributional heterogeneity and genetic structuring of mangrove populations, where gene flow is primarily in the form of sea water-dispersed propagules. Physical barriers to gene flow among mangrove populations include land masses, distance and direction of water and wind currents (Dodd et al., 2002; Nettel and Dodd, 2007; Triest, 2008; Van der Stocken et al., 2015). Mangrove distribution and population dynamics might be heavily influenced by historical sea-level fluctuations brought about by past climate changes (Woodroffe and Grindrod, 1991; Ellison, 2008), which would further reduce the long-term effective population size, in addition to genetic consequences of local extinction and recolonization associated with founder effects and genetic drift (McCauley, 1991). Postglacial range expansions can also induce the structuring of newly colonized areas into distinct sectors of low genetic diversity (Excoffier et al., 2009).

Life history traits have been shown to be closely associated with the amount of total genetic diversity at the species level and its partitioning within and among populations both in allozyme- and RAPD-based studies (Hamrick and Godt, 1989, 1996; Nybom and Bartish, 2000). Comparative studies of species with shared life history traits can help identify the major evolutionary forces shaping the patterns of genetic diversity. Although genetic diversity in some of the IWP *Rhizophora* species have been studied previously (e.g., Inomata et al., 2009; Wilson, 2009; Islam et al., 2013; Yahya et al., 2013; Wee et al., 2014, 2015), all these studies were limited to either one or two of the species (but see Ng et al., 2015), mostly covering only a small area of the species' distributional range. Furthermore, different number or type of molecular markers was used in different studies, rendering it difficult for comparative analysis of population genetic parameters across species between studies. To determine the level and pattern of genetic diversity in IWP species of *Rhizophora* and to provide information about their evolutionary history, we need to include all the *Rhizophora* species in the same study using the same set of markers from their entire distributional range. Furthermore, previous field work showed that there were two distinct leaf morphs within *R. apiculata*, separated by Wallace's line of discontinuity (Duke et al., 2002). Whether or not such discontinuity can be detected at the molecular level in *R. apiculata* as well as in *R. mucronata* and *R. stylosa* requires further studies. Phylogeographic investigation based on ITS and chloroplast DNA sequences showed that there were indeed two evolutionary lineages within each IWP *Rhizophora* species, largely corresponding to geographic separations of populations of SE Asia from those of Australia and Pacific islands (Lo et al., 2014). It remains to be investigated, however, whether population genetic distances based on allele frequencies at microsatellite loci can reflect this geographic pattern of discontinuity.

In this study, we used the same set of 13 microsatellite markers to investigate population genetic parameters for all three *Rhizophora* species occurring in the IWP region. Our samples covered these species' broad distributional ranges, including both central and peripheral populations. Specifically, we address the following questions: What is the level of genetic diversity in IWP *Rhizophora* species? Is the pattern of population structure the same among these species? Do populations from the species' distributional margins possess less genetic diversity than those central populations? Is the morphological and phylogeographic discontinuity still detectable with microsatellites in *R. apiculata* as well as in *R. mucronata* and *R. stylosa*? We then discuss the effects of life history traits and major evolutionary processes that might have played dominant roles in shaping the pattern of genetic diversity, as well as the significance of historical sea level fluctuations and physical barriers to dispersal on the genetic structuring of mangrove populations. We further discuss the effect of natural hybridization and genetic relationships among the three overlapping species. Our study is highly relevant to conservation and sustainable management of *Rhizophora* mangroves, such as to help identify management units, set priority for conservation, and provide guidance for translocations in mangrove reforestation practice.

## Materials and methods

### Plant samples

**Figure 1:**
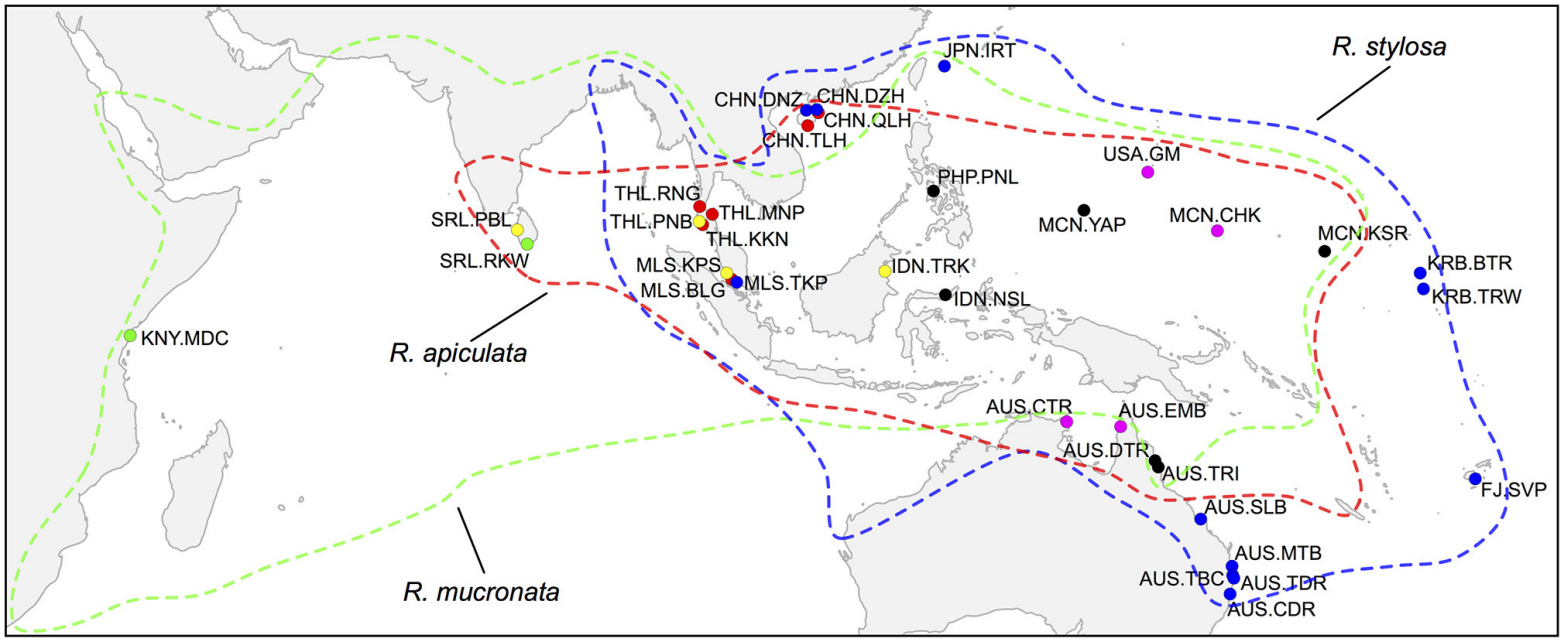
**Geographic locations of sampled populations of *Rhizophora* species in the Indo-West Pacific region**. Red, *R. apiculata*; green, *R. mucronata*; blue, *R. stylosa*. Yellow dots indicate sympatric sites where both *R. apiculata* and *R. mucronata* were sampled; magenta dots indicate sympatric sites where both *R. apiculata* and *R. stylosa* were sampled; black dots indicate sites where all three species were sampled. Colored lines represent the distributions of corresponding species.

To cover geographic distributions of the three *Rhizophora* species as wide as possible, we collected and genotyped a total 935 individuals from 54 populations across the whole IWP region (Table 1 and Figure 1). The present samples represent an expansion of those included in Lo et al. (2014), with much larger sample sizes at the population level for all three *Rhizophora* species. Fresh leaves were sampled from individual trees located at >10 m intervals to avoid non-random inclusion of closely related individuals within each wild population, dried with silica gel in sealed plastic bags, and preserved in an electronic auto-dry cabinet (Weifo, Taiwan).

**Table 1:** **Sampling locality information and estimates of genetic variation within populations of three *Rhizophora* species**.

**Country**	**Locality**	**Coordinates**	**Code**	***N***	***A***	***A*_R_**	***A*_E_**	***H*_O_ (SE)**	***H*_E_ (SE)**	***F*_IS_ (SE)**	***t***
***R. apiculata***											
China	Qinglan Harbor, Hainan	19°35'N/110°48'E	CHN.QLH	25	2.54	2.16	1.38	0.191 (0.047)	0.225 (0.055)	**0.173 (0.070)**	0.705
	Tielu Harbor, Hainan	18°16'N/109.42'E	CHN.TLH	25	2.23	1.95	1.41	0.249 (0.057)	0.251 (0.050)	0.028 (0.083)	0.946
Philippines	Panay Island	10°42'N/122°30'E	PHP.PNL	17	4.46	3.89	2.79	0.457 (0.090)	0.504 (0.081)	0.123 (0.089)	0.781
Micronesia	Chuuk	7°27'N/151°53'E	MCN.CHK	15	2.46	2.25	1.42	0.226 (0.050)	0.247 (0.053)	0.120 (0.115)	0.786
	Kosrae	5°17'N/162°58'E	MCN.KSR	19	2.23	1.94	1.32	0.190 (0.059)	0.191 (0.056)	0.033 (0.099)	0.936
	Yap	9°30'N/138°07'E	MCN.YAP	19	3.69	3.17	2.21	0.368 (0.067)	0.466 (0.065)	**0.235 (0.092)**	0.619
U.S.A	Guam	13°27'N/144°41'E	USA.GM	10	2.31	2.31	1.41	0.231 (0.065)	0.231 (0.055)	0.053 (0.093)	0.899
Malaysia	Blue Lagoon, Cape Rachado	2°24'N/101°51'E	MLS.BLG	20	3.38	2.96	2.02	0.515 (0.094)	0.414 (0.068)	−0.221 (0.076)	1.567
	Kampung Sementa, Klang	3°05'N/101°20'E	MLS.KPS	18	3.46	2.92	1.83	0.303 (0.063)	0.349 (0.070)	0.159 (0.074)	0.726
Sri Lanka	Pambala, Puttalam	7°32'N/79°50'E	SRL.PBL	22	2.85	2.39	1.54	0.196 (0.051)	0.292 (0.058)	**0.350 (0.117)**	0.481
Thailand	Khao Khanap Nam, Krabi	–	THL.KKN	17	3.38	2.88	1.82	0.308 (0.075)	0.335 (0.075)	0.111 (0.078)	0.800
	Ranong	–	THL.RNG	24	4.15	3.28	1.92	0.372 (0.065)	0.400 (0.064)	0.091 (0.074)	0.833
	Phang Nga Bay, Phunket	8°23'N/98°31'E	THL.PNB	27	4.15	3.16	2.02	0.353 (0.062)	0.405 (0.070)	0.146 (0.079)	0.745
	Mu Ko Thale Tai National Park	–	THL.MNP	16	4.54	4.09	2.82	0.342 (0.054)	0.533 (0.073)	**0.388 (0.089)**	0.441
Indonesia	North Sulawesi	1°22's/124°33'E	IDN.NSL	11	3.77	3.70	2.45	0.448 (0.081)	0.451 (0.083)	0.055 (0.044)	0.896
	Tarakan	–	IDN.TRK	6	3.31	NA	2.12	0.423 (0.079)	0.447 (0.068)	0.143 (0.117)	0.750
Australia	Cato River, Northern Territory	12°17's/136°21'E	AUS.CTR	21	3.15	2.56	1.58	0.282 (0.060)	0.286 (0.059)	0.038 (0.062)	0.927
	Embley River, Queensland	12°43's/142°02'E	AUS.EMB	11	2.54	2.50	1.90	0.378 (0.091)	0.379 (0.076)	0.050 (0.108)	0.905
	Daintree River, Queensland	–	AUS.DTR	26	2.46	1.92	1.21	0.124 (0.041)	0.139 (0.043)	0.125 (0.114)	0.778
	Trinity Inlet, Queensland	16°54's/145°46'E	AUS.TRI	13	1.85	1.78	1.19	0.136 (0.038)	0.138 (0.037)	0.055 (0.071)	0.896
***R. mucronata***											
Kenya	Mida Creek	–	KNY.MDC	13	2.69	2.59	1.90	0.290 (0.094)	0.316 (0.073)	0.121 (0.146)	0.784
Thailand	Phang Nga Bay, Phuket	8°23'N/98°30.5'E	THL.PNB	23	2.77	2.24	1.33	0.221 (0.072)	0.217 (0.040)	0.005 (0.114)	0.990
Micronesia	Yap	9°30'N/138°07'E	MCN.YAP	15	3.85	3.43	1.95	0.415 (0.054)	0.469 (0.029)	**0.148 (0.102)**	0.742
	Kosrae	5°17'N/162°58'E	MCN.KSR	8	2.92	NA	1.80	0.308 (0.087)	0.342 (0.072)	0.166 (0.132)	0.715
Indonesia	Tarakan	–	IDN.TRK	6	3.08	NA	2.11	0.359 (0.073)	0.466 (0.058)	**0.314 (0.114)**	0.522
	North Sulawesi	1°22's/124°33'E	IDN.NSL	13	4.08	3.75	1.99	0.432 (0.072)	0.445 (0.053)	0.069 (0.087)	0.871
Malaysia	Kampung Sementa, Klang	3°05'N/101°20'E	MLS.KPS	18	3.00	2.60	1.62	0.278 (0.086)	0.296 (0.063)	0.090 (0.125)	0.835
Philippines	Panay Island	10°42'N/122°30'E	PHP.PNL	22	4.31	3.35	1.93	0.280 (0.070)	0.370 (0.071)	**0.266 (0.112)**	0.580
Sri Lanka	Rekawa	–	SRL.RKW	8	1.92	NA	1.36	0.250 (0.085)	0.212 (0.055)	−0.113 (0.176)	1.255
	Pambala, Puttalam	7°32'N/79°50'E	SRL.PBL	12	2.46	2.34	1.56	0.321 (0.078)	0.310 (0.053)	0.009 (0.113)	0.982
Australia	Daintree River, Queensland	–	AUS.DTR	24	3.00	2.37	1.36	0.189 (0.072)	0.224 (0.045)	0.175 (0.141)	0.702
	Trinity Inlet, Queensland	16°54's/145°46'E	AUS.TRI	7	3.69	NA	2.56	0.330 (0.071)	0.583 (0.034)	**0.495 (0.112)**	0.338
***R. stylosa***											
Japan	Iriomote Island	24°35'N/123°47'E	JPN.IRT	24	2.85	2.27	1.43	0.252 (0.083)	0.239 (0.055)	−0.035 (0.132)	1.073
China	Danzhou, Hainan	19°51'N/109°33'E	CHN.DNZ	12	1.77	1.73	1.36	0.179 (0.076)	0.187 (0.061)	0.083 (0.189)	0.847
	Dongzhai Harbor, Hainan	19°55'N/110.37'E	CHN.DZH	21	2.00	1.82	1.36	0.216 (0.094)	0.177 (0.061)	−0.195 (0.135)	1.484
Philippines	Panay Island	10°42'N/122°30'E	PHP.PNL	18	3.23	2.91	1.96	0.248 (0.089)	0.356 (0.078)	**0.330 (0.137)**	0.504
Micronesia	Chuuk	7°27'N/151°53'E	MCN.CHK	20	4.00	3.23	2.11	0.338 (0.095)	0.392 (0.079)	**0.162 (0.128)**	0.721
	Kosrae	5°17'N/162°58'E	MCN.KSR	12	3.15	2.97	1.85	0.397 (0.089)	0.382 (0.065)	0.004 (0.126)	0.992
	Yap	9°30'N/138°07'E	MCN.YAP	20	2.62	2.29	1.54	0.288 (0.077)	0.295 (0.056)	0.047 (0.111)	0.910
U.S.A	Guam	13°27'N/144°41'E	USA.GM	17	1.77	1.68	1.28	0.199 (0.091)	0.149 (0.058)	−0.309 (0.140)	1.894
Malaysia	Teluk Pelanduk	2°25'N/101°53'E	MLS.TKP	17	3.08	2.43	1.38	0.267 (0.089)	0.205 (0.054)	−0.277 (0.060)	1.766
Indonesia	North Sulawesi	1°22's/124°33'E	IDN.NSL	17	3.77	3.24	2.01	0.448 (0.094)	0.404 (0.070)	−0.078 (0.095)	1.169
Kiribati	Butaritari Island	3°04'N/172°47'E	KRB.BTR	17	1.69	1.60	1.37	0.190 (0.093)	0.186 (0.067)	0.011 (0.275)	0.978
	Tarawa island	1°23'N/173°08'E	KRB.TRW	19	2.23	1.99	1.42	0.194 (0.084)	0.217 (0.064)	0.131 (0.178)	0.768
Fiji	Suva Point	18°09's/178°25'E	FJ.SVP	13	2.15	2.02	1.33	0.148 (0.073)	0.168 (0.058)	**0.157 (0.178)**	0.729
Australia	Cato River, Northern Territory	12°17's/136°21'E	AUS.CTR	23	6.15	5.08	3.66	0.645 (0.057)	0.650 (0.051)	0.029 (0.050)	0.944
	Embley River, Queensland	12°43's/142°02'E	AUS.EMB	16	5.23	4.65	3.04	0.630 (0.071)	0.600 (0.056)	−0.018 (0.057)	1.037
	Daintree River, Queensland	–	AUS.DTR	24	3.62	2.92	1.90	0.462 (0.073)	0.423 (0.056)	−0.070 (0.084)	1.151
	Trinity Inlet, Queensland	16°54's/145°46'E	AUS.TRI	14	3.00	2.75	1.73	0.418 (0.075)	0.370 (0.053)	−0.094 (0.085)	1.208
	Moreton Bay, Queensland	–	AUS.MTB	16	3.46	2.98	1.65	0.370 (0.073)	0.348 (0.052)	−0.033 (0.102)	1.068
	Shoalwater Bay, Queensland	22°20's/150°11'E	AUS.SLB	6	2.46	NA	1.87	0.487 (0.081)	0.415 (0.052)	−0.086 (0.118)	1.188
	Tallebudgera Creek, Queensland	28°06's/153°28'E	AUS.TBC	32	2.92	2.33	1.57	0.250 (0.071)	0.320 (0.049)	**0.233 (0.146)**	0.622
	Corindi River, New South Wales	29°58's/153°14'E	AUS.CDR	21	3.08	2.53	1.57	0.282 (0.081)	0.323 (0.048)	**0.150 (0.160)**	0.739
	Tweed River, New South Wales	28°09's/153°33'E	AUS.TDR	25	2.92	2.27	1.45	0.286 (0.087)	0.247 (0.059)	−0.139 (0.127)	1.323

*N, number of genotyped individuals in a population; A, average number of alleles; A_R_, rarefied allelic richness; A_E_, effective number of alleles; H_O_, observed heterozygosity; H_E_ expected heterozygosity; F_IS_, inbreeding coefficient; SE, standard error; N/A, not applicable due to small sample size (< 10). t, outcrossing rate, estimated as t = (1 − F_IS_)/(1 + F_IS_). Bold values in the F_IS_ column indicates significant heterozygosity deficit after Bonferroni correction at the significant level of 0.05*.

### Microsatellite genotyping

The dried leaf pieces of each individual sample were disrupted in a 2 ml microcentrifuge tube using QIAGEN TissueLyser II (QIAGEN, Hilden, Germany), and total genomic DNA was extracted using the standard CTAB method (Doyle and Doyle, 1987). Thirteen microsatellite loci, including RM11, RM38, RM41, and RM46 (Rosero-Galindo et al., 2002), Rhst01, Rhst02, Rhst11, Rhst13, Rhst15, and Rhst19 (Islam et al., 2004, 2013), RS19, RS59, and RS67 (Takayama et al., 2009), were genotyped for each sampled individual of all populations and species. Multiplex PCRs were carried out in a final volume of 5 μL with florescent-labeled primers using QIAGEN Multiplex PCR Kit (Qiagen, Hilden, Germany) following manufacturer's recommendation for microsatellite amplification. Specifically, the reaction mix contained 1 × QIAGEN Multiplex PCR Master Mix, 0.2 μM of each primer, and less than 0.1 μg template DNA. The PCR cycling protocol was as follows: an initial activation step for 15 min at 95°C, followed by 30 cycles of 30 s denaturation at 94°C, 90 s annealing at 57°C, and 60 s extension at 72°C, and a final extension step of 30 min at 60°C. Three multiplex mixes were applied in genotyping according to the compatibility of primers and the length of PCR product facilitated by Multiplex Manager 1.2 (Holleley and Geerts, 2009). RM11 (NED), RM38 (VIC), RM41 (6-FAM), and Rhst15 (PET) formed the first combination, RM46 (VIC), Rhst01 (6-FAM), Rhst02 (NED), and Rhst11 (PET) formed the second combination, and Rhst13 (VIC), Rhst19 (6-FAM), RS19 (6-FAM), RS59 (PET), and RS67 (NED) formed the last combination. The forward or reverse primer of each primer pair was labeled with a different dye (6FAM, Integrated DNA Technologies; NED, PET, and VIC, Applied Biosystems) as indicated in the parentheses following the locus name. PCR products were analyzed with an ABI 3130xl capillary sequencer following the manufacturer's instructions, with the GeneScan 500 LIZ size standard (Applied Biosystems) as internal marker. Alleles were scored using GENEMAPPER software version 4.0 (Applied Biosystems) and were manually checked for accuracy.

### Data analyses

Null alleles and potential stuttering were checked with MICRO-CHECKER version 2.2.3 (Van Oosterhout et al., 2004). The frequencies of null alleles were estimated with FREENA (Chapuis and Estoup, 2007) with 10,000 replications, using the implemented expectation maximization algorithm of Dempster et al. (1977). Linkage disequilibrium between each pair of loci across populations within each species was tested with FSTAT v2.9.3 (Goudet, 2001). No significant linkage disequilibrium was detected between loci in these populations.

Deviation from Hardy-Weinberg equilibrium (HWE) was tested for each of the locus—population combinations using GENEPOP (http://wbiomed.curtin.edu.au/genepop). The exact *p*-values of HWE tests were estimated with a Markov chain algorithm described by Guo and Thompson (1992), and the parameters of Markov chain were set as 10,000 dememorization steps and 1000 batches, with 10,000 iterations per batch. A global test of heterozygote deficiency was also carried out for each population using the same set of Markov chain parameters. The *p*-values of significance tests were subsequently adjusted for multiple tests with sequential Bonferroni correction (Holm, 1979; Rice, 1989).

The observed number of alleles per locus (*A*), rarefied number of alleles per locus (*A*_R_), and inbreeding coefficient (*F*_IS_) within populations were estimated using FSTAT 2.9.3 (Goudet, 2001). To reduce the bias arising from unequal population sample sizes, we used a standardized sample size of 10 to calculate *A*_R_ following the rarefication process of Petit et al. (1998). We further calculated the effective number of alleles per locus (*A*_E_) with GenAlEx version 6.5 (Peakall and Smouse, 2012), which corrects for uneven allele frequencies at each locus. The observed heterozygosity (*H*_O_) and expected heterozygosity (*H*_E_) within populations, and the total gene diversity (*H*_T_) at the species level, inbreeding coefficient within individuals (*F*_IS_) and genetic differentiation among populations (*F*_ST_) were also computed using GenAlEx version 6.5 (Peakall and Smouse, 2012). The multilocus outcrossing rate (*t*) was estimated based on the mean *F*_IS_ over loci within each population using the equation *t* = (1 − *F*_IS_)/(1 + *F*_IS_) (Wright, 1969). The estimate of *t* can exceed 1 or 100% if *F*_IS_ is negative, indicating heterozygote excess compared to random mating.

To analyze population genetic structure, we first carried out an allele-size permutation test to see whether mutation was non-negligible compared with genetic drift and migration (Hardy et al., 2003) before applying any *F*_ST_ estimation. We compared observed *R*_ST_values (before randomization) with *pR*_ST_ values obtained from 10,000 allele size permutations using SPAGeDi 1.4 (Hardy and Vekemans, 2002), and found no significant difference between them, confirming that mutation could not be the main cause of genetic differentiation among these populations. We then estimated *F*_ST_ (Weir, 1996) between all population pairs within each species and determined the significance of the estimate using FSTAT 2.9.3 (Goudet, 2001).

To further elucidate genetic relationships among populations at both intra- and interspecific levels, we performed the following three analyses. First, we conducted a discriminant analysis of principal components (DAPC, Jombart et al., 2010) for each species and for all three species combined using the R package adegenet (Jombart, 2008). We then examined genetic structure of each species and the relationships between species using the Bayesian model-based clustering method implemented in STRUCTURE v2.3.4 (Pritchard et al., 2000). Five independent assessments were carried out for each of the three species separately, for populations in all three species combined, and for populations of *R. mucronata* and *R. stylosa* combined. To determine the optimal *K*-value that best fits the data for each analysis, 20 iterations were performed for each *K*-value, from *K* = 1 to *K* = 10. Each run consisted of 2 × 10^6^ Markov chain Monte Carlo (MCMC) iterations with a burn-in period of 5 × 10^5^, employing the admixture model with correlated allele frequencies between clusters (Falush et al., 2003). Selection of the best *K*-value was done based on the Δ*K* parameter proposed by Evanno et al. (2005) and implemented in the Structure Harvester program version 0.6.94 (Earl and Vonholdt, 2012). For comparing the implied best *K*s and selected *K*s, 20 iterations were carried out with 2 × 10^6^ replicates of the MCMC after a burn-in period of 2 × 10^6^. We used the program CLUMPAK (Kopelman et al., 2015) to produce graphical displays. The third analysis was to characterize genetic relationships among populations using the neighbor-joining (NJ) method based on Nei's genetic distance (Nei et al., 1983) between populations with POPTREE2 (Takezaki et al., 2010), using a bootstrapping value of 10,000.

To estimate recent gene flow (*m*, proportion of individuals with migrant origin) between populations and between species, we used BayesAss v3.0.3 (Wilson and Rannala, 2003). The populations of this study were sampled to cover a vast geographic area of the three species' distributional ranges, but also included those from the same coastline and located adjacent to each other. In some locations, the same neighboring populations were sampled for more than one species. These population pairs provided rare opportunities for testing whether the direction of local oceanic current affected the direction of gene flow. Thus, we considered BayesAss analysis was well suited for detecting gene flow between closely located pairs of populations for comparison with the level of gene flow between those populations situated across vast stretches of oceans or on opposite sides of large land masses, in order to provide the *F*_ST_ estimates that reflect species-wide genetic differentiation among populations. We estimated recent gene flow between populations within each species with 3 × 10^8^ iterations, a burn-in value of 3 × 10^7^, and a sampling interval of 15,000. The mixing parameters for MCMC chains were adjusted in order to reach a swap acceptance rate between 20 and 60% as suggested by Wilson and Rannala (2003). The mixing parameters for estimating migration rate, inbreeding coefficient, and allele frequencies were set to 0.4, 1.0, and 0.8 respectively. Ten replicate runs of the MCMC with different seeds were carried out to check for convergence for each analysis. Visualization of chain mixing and convergence were accomplished with TRACER v1.6 (Rambaut et al., 2014). The results presented here were from the runs with the largest effective sample sizes.

## Results

### Genetic diversity

All the estimates of genetic diversity were relatively low at the population level in all three IWP *Rhizophora* species (Table 1), compared to the population mean values measured with microsatellite markers for other plant species (see Section Discussion below). The mean values of allelic richness were similar among the three species, such as the number of alleles per locus (*A*), averaged 0.315, 0.315, and 0.305 in *R. apiculata, R. mucronata*, and *R. stylosa* respectively, although the range of the estimates among populations was much broader, from 1.85 (AUS.TRI) to 4.54 (THL.MNP) in *R. apiculata*, from 1.92 (SRL.RKW) to 4.31 (PHP.PNL) in *R. mucronata*, and from 1.77 (CHN.DNZ) to 6.15 (AUS.CTR) in *R. stylosa*. At the species level, similar amount of total gene diversity (*H*_T_) was found in *R. apiculata* (0.621), *R. mucronata* (0.648), and *R. stylosa* (0.620) (Table 2), and nearly half of this diversity resided between populations, as indicated by the highly significant *F*_ST_estimates in all the three species.

**Table 2:** **Summary of genetic diversity at the species level**.

**Species**	***N*_ind_**	***N*_pop_**	***A***	***A*_E_**	***H*_O_**	***H*_S_**	***H*_T_**	***F*_IS_**	***F*_ST_**
*R. apiculata*	362	20	9.692 (1.298)	3.577 (0.533)	0.305 (0.045)	0.334 (0.045)	0.621 (0.070)	0.088 (0.060)	0.462^***^ (0.038)
*R. mucronata*	169	12	10.231 (1.063)	3.344 (0.542)	0.306 (0.060)	0.354 (0.035)	0.648 (0.052)	0.136 (0.120)	0.453^***^ (0.039)
*R. stylosa*	404	22	11.539 (1.289)	3.165 (0.391)	0.327 (0.067)	0.321 (0.038)	0.620 (0.050)	−0.021 (0.116)	0.483^***^ (0.046)

N_ind_, number of individuals; N_pop_, number of populations; A, number of different alleles; A_E_, number of effective alleles; H_O_, average observed heterozygosity across the populations; H_S_, expected heterozygosity within populations; H_T_, total gene diversity; F_IS_, inbreeding coefficient within individuals, F_IS_ = (Hs − H_O_)/ Hs. F_ST_, inbreeding coefficient within subpopulations, relative to total, i.e., genetic differentiation among populations, F_ST_ = (H_T_ − Hs)/ H_T_. Standard errors (SE, values in parenthesis) were estimated by jackknifing over loci. Stars (^***^) mean significant differentiation at the level of 0.001 based on 999 permutations.

Compared to populations located in or near the centers of these species' geographic ranges, the levels of genetic diversity were often lower in peripheral populations. For example, in *R. apiculata*, lower genetic diversity was found in populations located in its geographically margins, such as those in East Australia (AUS.DTR and AUS.TRI), China (CHN.QLH and CHN.TLH), and Pacific islands (USA.GM, MCN.CHK, and MCN.KSR; with a rare exception of MCN.YAP, *H*_E_ = 0.466), whereas relatively high estimates of allelic richness and heterozygosity were found in South East Asia, such as populations from Indonesia (IDN.NSL and IDN.TRK), Malaysia (MLS.BLG), Philippines (PHP.PNL), and Thailand (THL.RNG and THL.PNB). In *R. stylosa*, high levels of heterozygosity were found in two populations from North Australia (AUS.CTR, *H*_E_ = 0.650; and AUS.EMB, *H*_E_ = 0.600), whereas much lower levels were found in populations from China, Fiji, Guam, and Kiribati. However, there were exceptions to the general pattern, such as in *R. mucronata* the highest level of heterozygositywas found in East Australia (AUS.TRI, *H*_E_ = 0.583), while the lowest estimate was found in a population from Thailand (THL.PNB, *H*_E_ = 0.217).

Among a total of 702 locus-population combinations (calculated based on the 13 microsatellite loci × 54 populations studied), 164 of the combinations were monomorphic as some of the loci were not variable in all 54 populations. Significant deviations from HWE were detected in 30 of the remaining 538 locus-population combinations, and the global test of heterozygosity deficiency revealed that 13 out of 54 populations significantly deviated from HWE, corresponding to the significantly positive *F*_IS_ found in four populations (CHN.QLH, MCN.YAP, SRL.PBL, and THL.MNP) of *R. apiculata*, four populations (AUS.TRI, IDN.TRK, MCN.YAP, and PHP.PNL) of *R. mucronata*, and five populations (AUS.CDR, AUS.TBC, FJ.SVP, MCN.CHK, and PHP.PNL) of *R. stylosa* (Table 1). However, a correlation analysis of the estimated null allele frequencies (Supplementary Table 1) and *F*_IS_ values at each of the 13 microsatellite loci in these populations indicated that the presence of null alleles at some of the loci could result in an underestimate of *H*_O_ and thus an overestimate of *F*_IS_. We found that when null allele was not present at a locus, the estimate of *F*_IS_ was near zero or even negative, which indicates outcrossing. Because a species' mating system affects all loci equally, whereas null alleles affect only those loci where they are present, zero or negative *F*_IS_ at the loci without null alleles provide strong evidence for an outcrossing mating system in the same population. Thus, the multilocus outcrossing rate *t*, computed based on the mean *F*_IS_ over all loci (including those with null alleles), must represent an underestimate of outcrossing or an overestimate of inbreeding within each population. The estimates of *t* ranged from 0.441 to 1.567 among populations of *R. apiculata*, from 0.338 to 1.225 among populations of *R. mucronata*, and from 0.504 to 1.894 among populations of *R. stylosa*. The average outcrossing rate over populations was 0.821, 0.776, and 1.051 in *R. apiculata, R. mucronata*, and *R. stylosa*, respectively, indicating that they are predominantly outcrossing species, consistent with a primarily wind-pollinated mating system.

### Population genetic structure

Most of the intraspecific population pairs were significantly differentiated from each other (*P* < 0.05 after Bonferroni correction; Supplementary Table 2). However, the pairwise *F*_ST_ values were highly heterogeneous, ranging from 0.01 (between AUS.DTR and AUS.TRI) to 0.75 (between AUS.DTR and CHN.QLH) in *R. apiculata*; from 0.07 (MLS.KPS and THL.PNB) to 0.72 (AUS.DTR and SRL.RKW) in *R. mucronata*; and from 0.03 (AUS.TDR and AUS.MTB) to 0.76 (USA.GM and FJ.SVP) in *R. stylosa*. As expected, the high *F*_ST_ values were found between geographically distant populations, whereas the low *F*_ST_ values were found between geographically adjacent populations. At the species level, the mean *F*_ST_ values over all 13 loci were similar, 0.461 in *R. apiculata*, 0.452 in *R. mucronata*, and 0.484 in *R. stylosa* (Table 2). The high levels of population genetic differentiation were accordant with the presence of private alleles in many of the populations. Private alleles were detected in 9 of 20 populations of *R. apiculata*, 11 of 12 populations of *R. mucronata*, and 14 of 22 populations of *R. stylosa*. The number and frequencies of private alleles in these populations are given in Supplementary Table 3.

**Figure 2:**
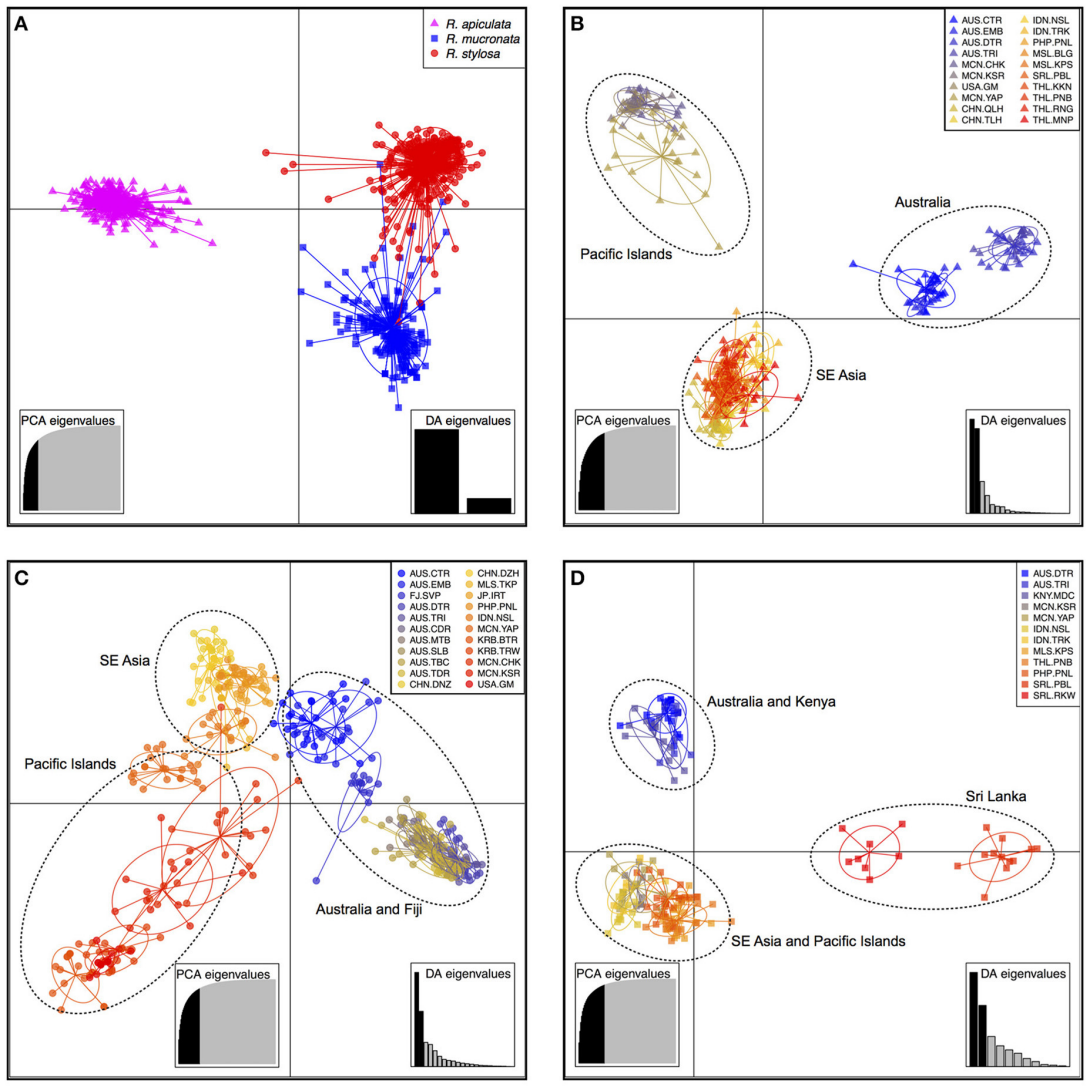
**Plots of discriminant analysis of principal components (DAPC) for all species combined (A), *R. apiculata* (B), *R. stylosa* (C), and *R. mucronata* (D)**. Species and populations are shown by inertia ellipses and different shapes and colors as indicated in the top-right inset of each figure, and dots represent individuals.

The DAPC results showed that individuals of *R. apiculata* could be clearly separated from those of *R. mucronata* and *R. stylosa* without ambiguity (Figure 2). However, the clusters of *R. mucronata* and *R. stylosa* showed some degree of overlapping (Figure 2A). Three clusters were identified in *R. apiculata*, namely Australia, Pacific islands, and SE Asia (Figure 2B). This pattern was also detected in *R. stylosa*, but the separation between the three geographical groups was not as well-defined as in *R. apiculata* (Figure 2C). In *R. mucronata*, individuals from Australia and Kenya were grouped together, individuals from SE Asia and Pacific islands were grouped together, and individuals from Sri Lanka were separated from the other two clusters (Figure 2D).

**Figure 3:**
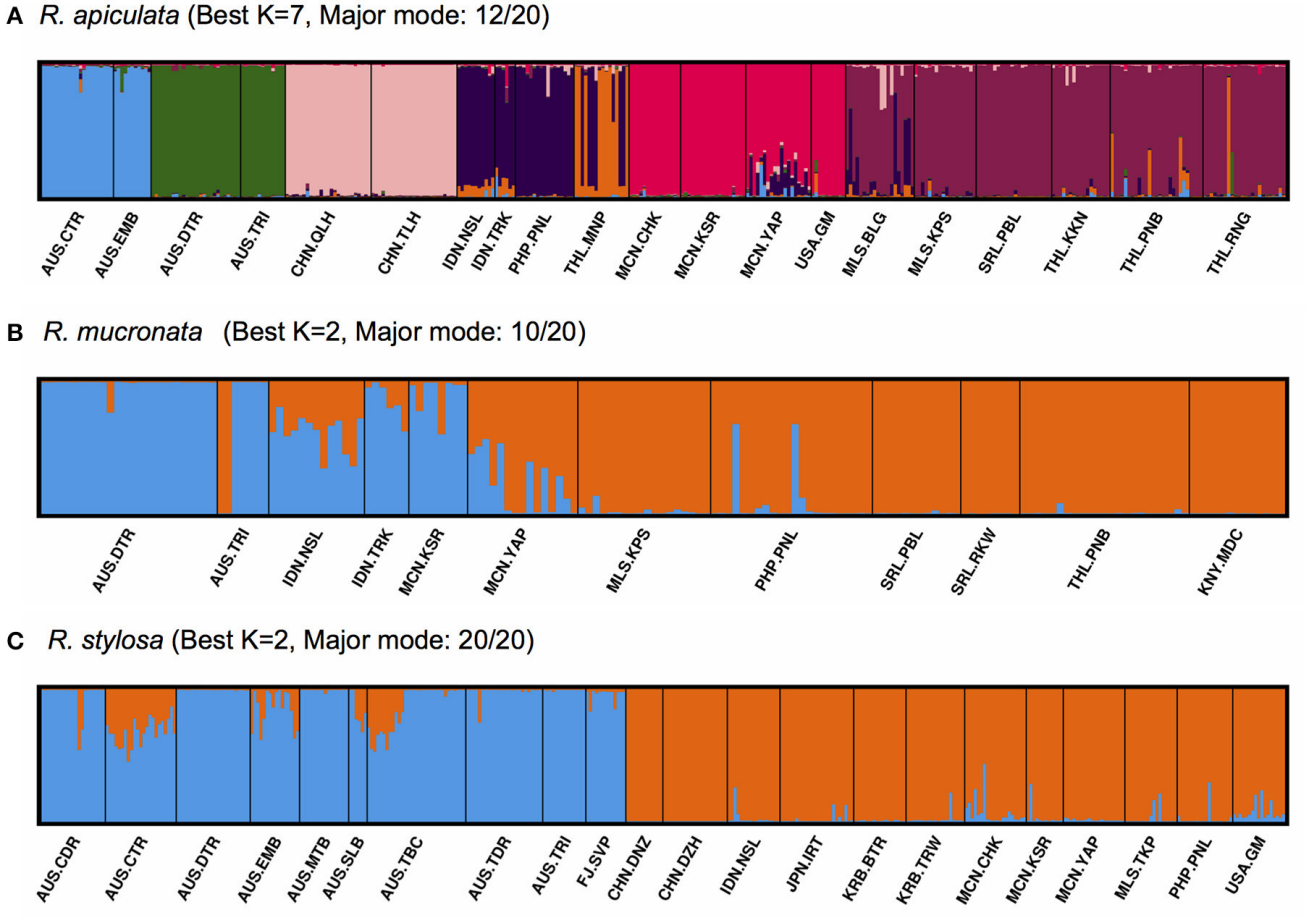
**Structure bar plots showing the assignment of individuals in *Rhizophora***. Each vertical bar represents one individual, and different colors within each bar indicate admixture. **(A)** *R. apiculata* (best *K* = 7); **(B)** *R. mucronata* (best *K* = 2); **(C)** *R. stylosa* (best *K* = 2).

The inferred best *K*-values from STRUCTURE cluster analysis (Figure 3; Supplementary Figure 1) were different between the species, with *K* = 7 clusters for *R. apiculata* (Figure 3A), 2 clusters for *R. mucronata* (Figure 3B), and 2 for *R. stylosa* (Figure 3C). When adopting the same *K* = 2 clusters for interspecific comparisons, populations of *R. apiculata* from Australia and Pacific Islands formed one cluster, and all populations from SE Asia formed the other (Supplementary Figure 2A). The minor mode of *K* = 7 in *R. apiculata* (supported by 7 out of 20 iterations; Supplementary Figure 2B) was similar to the major mode (12 out of 20; Figure 3A), but with all Australian populations placed in one group. In *R. mucronata*, the major cluster mode (10 out of 20; Figure 3B) separated most of the populations from Australia, Indonesia, and Micronesia from others, but with considerable admixture in several of the populations. However, a minor cluster mode (7 out of 20; Supplementary Figure 2C) separated the two Australian populations from all others, whereas the second minor mode (3 out of 20; Supplementary Figure 2D) placed populations from Australia, Sri Lanka and Kenya into one group, and all other populations formed the second cluster. In contrast, populations of *R. stylosa* from Australia and Fiji were well separated from populations of SE Asia and Pacific Islands, with little admixture.

**Figure 4:**
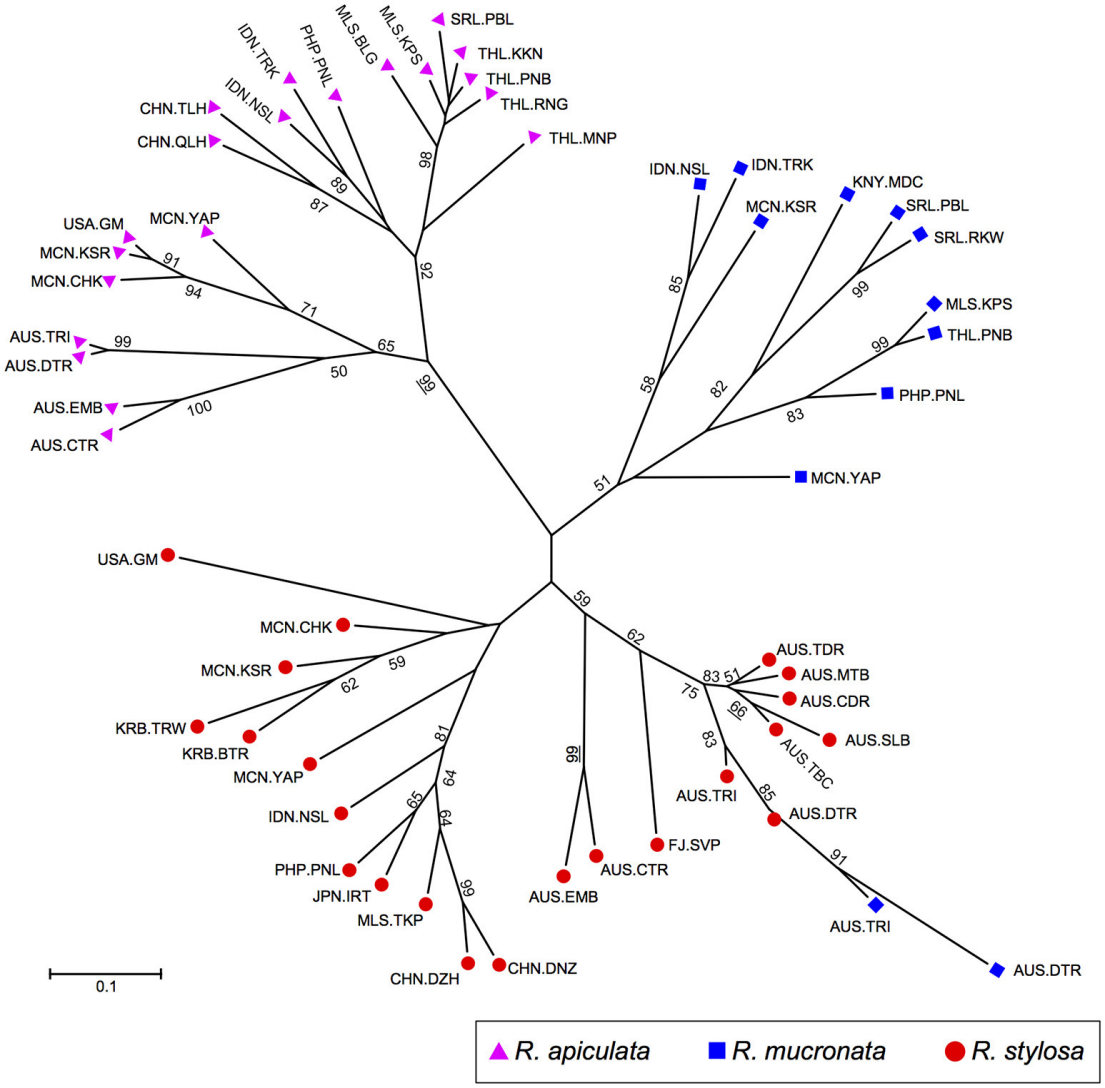
**Neighbor-joining tree based on Nei's genetic distance showing relationships among *Rhizophora* populations**. Purple triangles, *R. apiculata*; Blue squares, *R. mucronata*; and red circles, *R. stylosa*.

The NJ analysis based on allele frequencies in populations of all three species showed three major groups (Figure 4), with each group consisting of largely conspecific populations. Within the *R. apiculata* group, populations can be further divided into two subgroups, one consisting of populations from South East Asia, and the other including populations form Australia and Pacific islands. Populations of *R. mucronata* were grouped together with two exceptions, AUS.TRI and AUS.DTR from Australia, which were grouped with populations of *R. stylosa* from the sympatric sites. The *R. stylosa* group was further divided into two subgroups, with populations from Pacific islands and South East Asia forming one group and all Australian populations and Fiji forming the other. Among the Australian populations, the two populations from North Australia (AUS.EMB and AUS.CTR) were clearly divergent from those of East Coast.

### Recent gene flow

The estimates of gene flow/migration rate between intraspecific populations, ranged from 0.0068 to 0.1721 in *R. apiculata*; from 0.0088 to 0.2052 in *R. mucronata*, and from 0.0062 to 0.1513 in *R. stylosa* (Supplementary Table 4). With rare exceptions, the relatively higher rate of gene flow was detected for geographically adjacent population pairs, e.g., from AUS.TRI to AUS.DTR (0.1389) and from THL.RNG to THL.PNB (0.1721) in *R. apiculata*; from AUS.TRI to AUS.DTR (0.0866) and from SRL.RKW to SRL.PBL (0.1425) in *R. mucronata*; from AUS.EMB to AUS.CTR (0.1419) and from AUS.CDR to AUS.TDR (0.1513) in *R. stylosa*. Furthermore, strong directional differences in gene flow were detected between some of these adjacent populations, e.g., gene flow from AUS.EMB to AUS.CTR was much higher than that from the opposite direction in both *R. apiculata* (0.1285 vs. 0.0077) and *R. stylosa* (0.1419 vs. 0.0072); gene flow from AUS.TRI to AUS.DTR was up to 18 times higher than that from AUS.DTR to AUS.TRI and the same pattern of directionality was observed in all three species (0.1389 vs. 0.0072 in *R. apiculata*; 0.0888 vs. 0.0089 in *R. mucronata*; and 0.1243 vs. 0.0070 in *R. stylosa*). Strong directional differences in gene flow were detected in seven population pairs in *R. apiculata*, four in *R. mucronata*, and seven in *R. stylosa* (highlighted in the Supplementary Table 4), suggesting that directionality of wind and ocean currents must have played a major role in the dispersal of water-floating propagules or pollen between adjacent populations of *Rhizophora*.

## Discussion

The present levels of genetic diversity and population structure in mangrove tree species, which border on the terrestrial edge with ocean-current dispersal, are the consequences of their unique evolutionary history, environmental factors, and life history traits. It is often challenging to untangle the relative importance of these factors or their interactions in determining population genetic characteristics. Comparative analysis of closely related species with shared life history traits and similar ecological and environmental conditions could greatly reduce the difficulties of interpretation by reducing the number of variables arising from different evolutionary pathways. In the present study, we included all three IWP *Rhizophora* species to investigate the major evolutionary processes and environmental factors in shaping the pattern of genetic diversity and population structure in these ecologically and economically important mangrove tree species.

### Pattern of genetic diversity

Comparative analyses of allelic diversity, observed and expected heterozygosities revealed similarly low genetic variation at the population and species levels in the three IWP *Rhizophora* species, which is consistent with their common life history traits, similar environmental conditions and evolutionary history. However, in contrast to the expectation that long-lived woody species maintain more variation within species and within populations but have less variation among populations than species with other life forms (Hamrick et al., 1992), we detected an opposite pattern of population genetic diversity in the three *Rhizophora* species: mostly low variation within populations but high differentiation among populations. Compared to the mean values of genetic variation at the population level estimated with genomic microsatellite markers in species belonging to the same taxonomic group rosids of eudicots (*A* = 6.21, *H*_O_ = 0.480, and *H*_E_ = 0.579; Merritt et al., 2015), all the corresponding measures of genetic variation were much lower in the populations of IWP *Rhizophora* (Table 1), despite being primarily wind-pollinated, long-lived trees with ocean current-dispersed seedlings and wide geographic distributions. We considered possible sampling effects, but did not find any significant relationships between population sample size and genetic diversity parameters (correlation test *p* = 0.276 and 0.145 for *A* and *H*_E_, respectively). Repeated founder effects or bottleneck events associated with geo-climatic history in the IWP region and genetic drift in small and geographically isolated populations are most likely the major cause of the low genetic diversity within populations and within species of *Rhizophora*, especially in marginal populations of the species' distribution ranges. With rare exceptions, the lowest estimates of genetic diversity were found in the peripheral populations of each species. Low genetic diversity in marginal populations is also shown in other studies of *R. stylosa*. For example, Wilson (2009) sampled 20 populations of *R. stylosa* from eastern Australia (representing the species' southern geographic range limit), and reported much lower estimates of heterozygosity (*H*_O_ = 0.19 and *H*_E_ = 0.26). Islam et al. (2013) reported a very low level of *H*_O_ (0.130) but higher *H*_E_ (0.338) in 16 populations of *R. stylosa* from Sakishima islands of the Japanese archipelago (representing the species' northern distributional edge). These marginal populations of *Rhizophora* may represent descendants of a few ancestral propagules dispersed from the more centrally located populations after the last glacial maximum (LGM), resulting in further reduction of genetic variation due to the founder effect. Low genetic diversity in peripheral populations was also detected in other mangrove tree species, such as *Bruguiera gymnorhiza* (Rhizophoraceae) from its northern geographic limit in Japan (Islam et al., 2014), and *Rhizophora mangle* and *Avicennia germinans* (Avicenniaceae) in the AEP region (Pil et al., 2011; Sandoval-Castro and Muñiz-Salazar, 2012; Sandoval-Castro et al., 2014).

Geographic distributions of mangrove trees are strongly influenced by air temperature and availability of intertidal habitats, with the latter being tightly linked to sea level fluctuations (Duke et al., 1998). During the Pleistocene epoch, the sea level fluctuated to a great extent, up to 125 m lower than the present level (Voris, 2000). Along with every cycle of warming and cooling, mangrove populations, especially those located in their distributional limits, would have experienced repeated local extinction and recolonization by a few founders, leading to a very small long-term effective population size (*N*_E_), which is often a small fraction of the census population size (Woodruff, 2001; Frankham et al., 2002). The generally small *N*_E_ alone could account for the observed pattern of low variation within populations and high divergence between populations due to enhanced genetic drift in mangroves. A recent large-scale comparative genomic study has shown that mangrove populations generally have much smaller *N*_E_ and lower level of heterozygosity than non-mangrove plants based on genome-wide single nucleotide polymorphism (SNP) data (S. Shi and C-I Wu, personal communications). It can be concluded that genetic drift might have played a dominant role in the evolutionary history of mangrove trees, as evidenced at both SNP and microsatellite loci across the genome.

Compared to the present estimates, much lower levels of allelic diversity and heterozygosity in populations of *R. mucronata* and *R. stylosa* (e.g., *H*_O_ averaged 0.108 and 0.097 respectively) were reported in Wee et al. (2015). They interpreted the low heterozygosity as a result of inbreeding in the two species. However, other factors need to be taken into consideration before attributing the lack of heterozygosity to inbreeding. Furthermore, the characteristics of microsatellite markers can affect the estimates of genetic variation (Merritt et al., 2015). For comparing the level of genetic variation between species or between studies, it is necessary to use the same set of marker loci. Nevertheless, despite the difference in marker loci employed between studies, the same pattern of genetic diversity: low within- but high between-population variation, was found in *R. mucronata* and *R. stylosa* in this study and Wee et al. (2015) as well as in *R. apiculata*, indicating that the same evolutionary processes operated on genetic diversity in these species.

### Inbreeding coefficient, outcrossing rate, and the effect of breeding system on genetic diversity

*Rhizophora* species are self-compatible, weakly protandrous, and predominantly wind-pollinated, though pollination by small insects may also exist (Tomlinson, 1986; Kondo et al., 1987; Nadia and Machado, 2014). According to their population outcrossing rates (*t*), self-compatible plant species can be further characterized as predominantly outcrossing (*t* > 80%), mixed mating (*t* = 20–80%), or predominantly selfing (*t* = 0−20%). Although *t* is commonly computed using progeny arrays (e.g., Sun and Ganders, 1986, 1988, 1990; Sun and Ritland, 1998; Sun et al., 1998), it also can be indirectly estimated based on Wright's within-population inbreeding coefficient (Wright, 1965, 1969) using the relationship *t* = (1 − *F*_IS_)/(1 + *F*_IS_) as described by Weir (1996). Our present estimates of *t* based on *F*_IS_ were similarly high in *R. apiculata* and *R. mucronata*, and the estimates were still higher for populations of *R. stylosa*. Accordingly, the breeding systems of these species can be defined as mixed mating or predominantly outcrossing. Given that the population *F*_IS_ value might represent an overestimate of inbreeding if null alleles are present at some of the marker loci, the estimate of *t* could even be higher. Hence, a shift in mating system toward inbreeding is unlikely a valid explanation for the generally low levels of genetic variation within their populations.

Several previous studies reported various levels of inbreeding in populations of *Rhizophora* from both IWP and AEP regions (e.g., Lowenfeld and Klekowski, 1992; Dominguez et al., 1998; Cerón-Souza et al., 2012; Yahya et al., 2013), as well as in populations of other mangrove species, such as *Avicennia marina* (Maguire et al., 2000), *Ceriops decandra* (Tan et al., 2005), and *Kandelia candel* (Chen et al., 1996; Sun et al., 1998). For example, field observations of bee activities suggested that there might be substantial geitonogamous selfing in populations of *K. candel* and thus quantitative analysis of the mating system parameters was performed using progeny arrays assayed for allozyme markers (Sun et al., 1998). Although the breeding system of *K. candel* can be regarded as mixed-mating based on the estimated outcrossing rate in two of its populations (0.697 ± 0.091 and 0.797 ± 0.062 respectively), the levels of allozyme variation were extremely low at both population and species levels in comparison with other plant species with mixed-mating system. Random genetic drift and founder effects are most likely the driving force in mangrove populations, especially in the species' distributional margins, overriding the effect of breeding system on the level and pattern of genetic diversity.

With rare exceptions (e.g., Lowenfeld and Klekowski, 1992; Sun et al., 1998), the estimates of *F*_IS_ have been commonly used to infer inbreeding in many mangrove studies. This is probably due to the difficulties to assay progeny arrays with mangrove propagules. However, microsatellite-based estimates of *F*_IS_ should be interpreted with caution if null alleles are present at one or more of the sampled loci, because null alleles could result in an excess of observed homozygosity compared to HWE expectation and consequently result in an overestimate of *F*_IS_ at the locus. Null alleles are commonly present at microsatellite loci (Chapuis and Estoup, 2007), thus the estimates of *F*_IS_ at each locus should be carefully checked before inferring inbreeding at the population level.

In this study, we also detected significant *F*_IS_ in four of the 20 populations of *R. apiculata*, four of the 12 populations of *R. mucronata*, and five of the 22 populations of *R. stylosa*. After carefully examining the estimates of *F*_IS_ at each locus in these 13 populations, we found that the deficiencies of observed heterozygosity in all the populations with significant *F*_IS_ could be explained by the presence of null alleles at some of the marker loci in these populations, because other loci in the same individuals had nearly zero or even negative *F*_IS_. Because a species' mating system affects all genetic loci equally, whereas null alleles affect only those loci where they are present, thus near zero or negative *F*_IS_ at some of the individual loci provides stronger evidence for random mating or outcrossing than any high values of positive *F*_IS_ at different loci in the same population as evidence for inbreeding. We think heterogeneity in *F*_IS_ across the sampled loci within the same population, rather than the mean value of *F*_IS_ over these loci, should be taken into account before inferring inbreeding at the whole population level.

**Figure 5:**
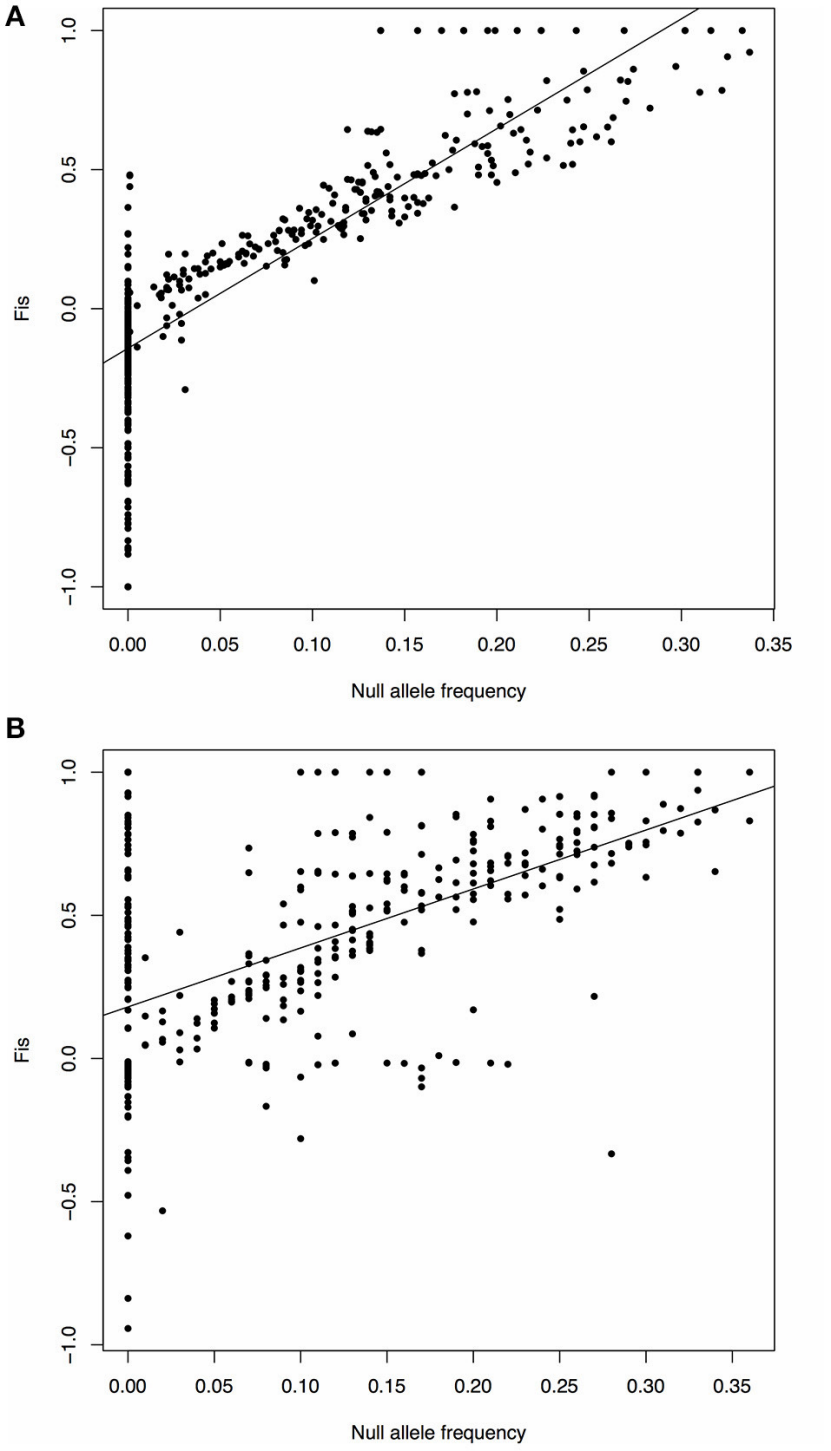
**Scatter plot and fitting linear regression of null allele frequency and *F*_IS_**. The X axis represents the null allele frequency, and the Y axis represents the *F*_IS_ value. The black line shows the fitting linear regression. **(A)** Data from this study. *Y* = −0.1425+3.9492X; *P* < 2.2e^−16^; the correlation coefficient *r* = 0.8493. **(B)** Data from Wee et al. (2015). *Y* = 0.1802+2.0604X; *P* < 2.2e^−16^; *r* = 0.5622.

We found that *F*_IS_ values were highly heterogeneous among the marker loci in the 13 populations with significant *F*_IS_, suggesting that the significant *F*_IS_ values were likely caused by the presence of null alleles at some of the loci in these populations, rather than reflecting the actual population level of inbreeding. To prove this might be the case, we further analyzed the relationship between null allele frequencies and *F*_IS_ estimates at each of the sampled loci for all populations included in the present study, and also did the same analysis for the data given in Wee et al. (2015; original data from http://datadryad.org/resource/doi:10.5061/dryad.42711/1). We found highly significant correlations (*p* < 2.2e^−16^) between the two estimates in both of the studies (Figure 5). This relationship indicates caution should be exercised in inferring inbreeding from microsatellite-based *F*_IS_ estimates in all future population genetic studies. Because it is often impractical to eliminate all the loci with null alleles if they are present in only a few sampled populations but not in most others, a simple solution to this problem is to inspect heterogeneity in the *F*_IS_ estimates across all sampled loci for each population, as we have done here in data analysis and interpretation.

### Gene flow and population genetic differentiation

We detected a generally low level of gene flow between pairs of populations within each *Rhizophora* species, as most of these populations were separated by a vast body of sea water or land barriers. In contrast, gene flow can be relatively high between neighboring populations along the same coastline. We also detected a strong directional asymmetry in the level of gene flow between some of these adjacent populations. For example, the direction of gene flow was predominantly northward between AUS.TRI and AUS.DTR, and westward between AUS.EMB and AUS.CTR; and this same pattern of directionality was detected in all the three *Rhizophora* species. Therefore, to fully understand the levels of gene flow and pattern of population genetic differentiation, the simple “isolation by distance” model alone may not be applicable for mangrove species. Other factors, such as directions of wind and local current, costal topographies as well as land barriers need to be considered in addition to geographic distances between populations.

High levels of gene flow and hence low genetic differentiation between populations are often associated with long-lived trees with predominant wind pollination and water-dispersed seeds. But this is not the case in all the three IWP *Rhizophora* species. Despite that their large water-buoyant propagules could preserve viability after floating for long periods of time in salt water (Davis, 1940; Rabinowitz, 1978; Drexler, 2001), the presumed capacity for long-distance dispersal via ocean currents was not supported in the present study, as indicated by the generally low level of gene flow and high genetic differentiation among their populations. Wee et al. (2014, 2015) also found similarly high levels of population genetic differentiation in *R. mucronata* and *R. stylosa*. Other physical factors, such as estuarine geomorphology and the interplay between water and wind currents likely played important roles in facilitating or blocking gene flow. For instance, Van der Stocken et al. (2015) showed that whether mangrove propagules could reach the open ocean depends on the strength and direction of both outgoing water flow and winds. Other studies have found that the dispersal of *Rhizophora* seedlings was mostly restricted to narrow geographic scales (Cerón-Souza et al., 2012; Islam et al., 2013). High levels of genetic differentiation were also found in other water-dispersed mangrove species, including *Avicennia* (Maguire et al., 2000; Dodd and Rafii, 2002), *Ceriops tagal* (Ge and Sun, 2001; Huang et al., 2012), and *B. gymnorhiza* (Minobe et al., 2009). Land barriers, vast oceanic surface distance, and directionality and dynamic interactions of wind and current, whether acting alone or in synergy, apparently played important role in determining the levels of gene flow between populations in most of the mangrove species.

Although the presence of null alleles may lead to an overestimation of genetic differentiation (Chapuis and Estoup, 2007), the effects could be negligible if they were uncommon or rare, i.e., at a frequency of < 0.2 (Carlsson, 2008; Queloz et al., 2010). The MICRO-CHECKER results showed that null alleles were not present at any of the 13 microsatellite loci in 16 populations, and the estimated null allele frequencies in the remaining 38 populations were generally low, only 45 out of 702 locus-population combinations in excess of 0.2. Because none of the null alleles at any of the microsatellite loci was commonly present in these populations, all the 13 loci were used in our data analyses. Still the possible effects of null alleles on *F*_ST_ estimates should be taken into account. Thus, we compared the *F*_ST_ estimates from FSTAT (null alleles included) with those from FREENA (null alleles excluded). We found that the estimates of genetic differentiation were comparably high whether or not the null alleles were included in the data analyses (Supplementary Table 2).

It should be noted, however, the estimates of genetic differentiation between populations are also found to be closely correlated with maximum geographic distance between the analyzed populations in outcrossing species (Nybom and Bartish, 2000). This indicates the importance of sampling strategies in population genetic studies. Limited coverage of the species' geographic distribution in population sampling could potentially bias *F*_ST_ estimates downward. Thus, geographic range of the samples should closely match the species' distributional range in the analysis of population genetic structure, as we have done in the present study.

### Genetic divergence and phylogeographic disjunction

At individual species level, genetic divergence among populations may not follow the same geographic pattern. In this study, the STRUCTURE analysis showed that populations of *R. apiculata* were highly divergent, forming 7 small clusters among the 20 sampled populations. However, only two main clusters were present in the neighbor-joining analysis of genetic distances among these populations, showing a deep division between Australasian and Indo-Malaysian populations, coincident with the marine Wallace's Line (Wallace, 1863). This pattern of two notable clusters of populations of *R. apiculata* is concordant with its phylogeographic disjunction previously revealed based on morphological traits (Duke et al., 2002) and cpDNA and ITS sequences (Lo et al., 2014), suggesting the effect of ancient separation of Australia and SE Asia geological plates. Subsequent genetic differentiation between the Pacific island populations and those of Australia could be caused by the rarity of long-distance dispersal and hence lack of subsequent gene flow following the initial colonization of the islands from source populations in Australia. Within SE Asia, populations on the western side of Malay Peninsula were separable from those on the eastern side, suggesting that the Malay Peninsula acted as a land barrier to the dispersal of *R. apiculata*, which is consistent with previous studies on *Rhizophora* species (Inomata et al., 2009; Ng et al., 2015; Wee et al., 2015). This pattern of population differentiation is also evident in other sea water dispersed mangrove species, such as *Ceriops* species (Ge and Sun, 2001; Liao et al., 2007; Huang et al., 2008, 2012), *B. gymnorhiza* (Minobe et al., 2009; Urashi et al., 2013), and *Lumnitzera* species (Su et al., 2006, 2007; Li et al., 2016). Similarly, genetic structure of *R. mangle, R. racemosa*, and *R. samoensis* in the AEP region is influenced by the Central American Isthmus and the American continent land masses (Núñez-Farfán et al., 2002; Takayama et al., 2013).

Genetic divergence among populations of *R. mucronata* and *R. stylosa* did not exhibit the same pattern as seen in *R. apiculata*. All the Australian populations of both *R. mucronata* and *R. stylosa* were still well separated from those of SE Asia, but their Pacific island populations were more similar to SE Asian than to Australian populations, as reflected by DAPC, STRUCTURE, and NJ analyses. This pattern of population genetic divergence could not be explained by ancient vicariant events, which were expected to affect co-distributed taxa simultaneously and thus produce similar patterns of phylogeographic disjunction, as revealed previously in both *R. apiculata* and *R. mucronata/stylosa* in the IWP region with DNA sequence data (Lo et al., 2014). The incongruity between population genetic divergence and phylogeographic disjunction might arise from the difference in the rate of evolution at different molecular marker loci. Microsatellites evolve much faster than DNA sequences, especially faster than cpDNA sequences in plant species (Freeland et al., 2011). Rosenbaum and Deinard (1998) have pointed out some problems with attempting to resolve evolutionary relationships between taxa or among intraspecific populations through microsatellite analysis. Nevertheless, microsatellites continue to be the marker of choice in a wide variety of studies, especially for investigation of population genetic structure and paternity analysis (Merritt et al., 2015).

### Natural hybridization between species

Our STRUCTURE and gene flow analyses of combined populations (including two or all three species together) showed that *R. apiculata* was indeed distinct from *R. mucronata* and *R. stylosa*, but the species boundary between the latter two taxa was blurry (Supplementary Figures 3, 4). For example, in the combined STRUCTURE analysis including both *R. mucronata* and *R. stylosa*, the Australian populations AUS.TRI and AUS.DTR of *R. mucronata* were grouped together with those of *R. stylosa* from the same sites (Supplementary Figure 3), similar to the finding based on NJ analysis (Figure 4). These findings are congruent with the extent of natural hybridization as well as phylogenetic relationships among the three species. Natural hybridization between *R. apiculata* and *R. stylosa* or *R. mucronata* resulted in sterile F1 hybrids, named as *R*. x *lamarckii* or *R. annamalayana* (e.g., Kathiresan, 1995; Chan, 1996; Ng et al., 2013). These hybrid sterilities indicate a strong postzygotic isolation mechanism maintaining morphological and genetic distinctions between sympatric *R. apiculata* and *R. mucronata/R. stylosa*. In contrast, *R. mucronata* and *R. stylosa* are morphologically similar and difficult to distinguish even by experts in the field (Duke et al., 2002). When all 54 populations of the three species were combined in the same analysis, the level of gene flow between *R. mucronata* and *R. stylosa* was many times higher than between *R. mucronata/R. stylosa* and *R. apiculata* (Supplementary Figure 4). However, no putative hybrids between *R. mucronata* and *R. stylosa* have been described based on morphological traits, as the differences between the parental species are subtle and the identification of morphologically intermediate individuals is nearly impossible. Ng and Szmidt (2015) provided genetic evidence for substantial introgression between *R. mucronata* and *R. stylosa* at all studied locations where they co-occur, indicating the absence of reproductive barrier. Without pre- or postzygotic barriers, interbreeding between *R. mucronata* and *R. stylosa* would be equivalent to gene flow within the same species. Any existing morphological or genetic divergence between the two parental taxa, in the absence of strong local selection for their maintenance, would tend to disappear at the sites of sympatry. This could explain the difficulties in separating *R. mucronata* from *R. stylosa* in the two Australian populations AUS.TRI and AUS.DTR where they coexist.

Despite the difficulties in distinguishing *R. mucronata* and *R. stylosa* based on morphological keys (such as leaf size and length of flower styles; Duke et al., 2002), there seems to be differential habitat preference: *R. stylosa* frequently occurs in hard sandy soil and more exposed offshore sites, whereas *R. mucronata* occurs mostly in areas subject to regular freshwater flows, at least in the eastern part of its range (Duke, 2006). These observations suggest that environmental conditions might have exerted heavy influence on the morphological characters used to delimit *R. stylosa* and *R. mucronata*. Also, the morphological or genetic divergence between the two taxa likely developed independently in multiple localities, resulting in polyphyletic origins of both *R. mucronata* and *R. stylosa* (Lo et al., 2014). Thus, it seems appropriate to consider *R. mucronata* and *R. stylosa* as different ecotypes of the same species, or at most as incipient ecological species, assuming that the speciation process is not reversed due to ongoing hybridization/gene flow. Genome wide SNP markers can be explored to shed further light on the pattern of genetic divergence, hybridization, and introgression between populations of *R. mucronata* and *R. stylosa*.

## Conclusion

*Rhizophora* species are the most widely distributed mangrove trees in the IWP region. This study represents the first comprehensive, large-scale population genetic investigation that included all three species of IWP *Rhizophora* across their distributional ranges. Although long-lived woody trees with predominant wind-pollination, water-dispersed seeds, and broad geographical range are expected to maintain more genetic variation within populations and less genetic differentiation between populations than species with other life history traits, an opposite pattern of genetic diversity was revealed in *R. apiculata, R. mucronata* as well as *R. stylosa*: all estimates of within-population genetic variation were very low and between-population genetic differentiation was high. This pattern of genetic diversity indicates genetic drift coupled with limited gene flow likely played the dominant role in the evolutionary history of mangrove trees, as frequent sea level fluctuations associated with climate changes would negatively impact their effective population sizes and various physical barriers hinder long-distance dispersal of their propagules. Our findings indicate that, in addition to life history traits, historical, demographical, ecological, and evolutionary factors must be taken into account to understand the organization of genetic variation among individuals both within and between populations. Furthermore, we discussed the genetic relationships among the three *Rhizophora* species, hybrid sterility barriers effectively maintain morphological and genetic distinction of *R. apiculata* at the sites of sympatry, but *R. mucronata* and *R. stylosa* are morphologically and genetically similar and lack reproductive isolation, suggesting that they could be treated as ecotypes of the same species.

## Author contributions

MS initiated the research; ND collected most of population samples; YY performed experiments; YY and MS analyzed data and wrote the paper.

## Funding

This research was funded by a HKRGC grant (HKU7261/00M) and a CRCG grant to MS, and a postgraduate fellowship from the University of Hong Kong to YY.

### Conflict of interest statement

The authors declare that the research was conducted in the absence of any commercial or financial relationships that could be construed as a potential conflict of interest.
