# Supplementary material for: Comparative Analysis of the Pattern of Population Genetic Diversity in Three Indo-West Pacific Rhizophora Mangrove Species
Source: Front Plant Sci. 2016 Sep 30;7:1434. doi: 10.3389/fpls.2016.01434 (PMC5043064; doi:10.3389/fpls.2016.01434)
Supplement: Supplementary file 1 [file DataSheet1.DOCX]

Supplementary Material

**Comparative Analyses of Population Genetic Structure in Three *Rhizophora* Species from the Indo-West Pacific Region**

**Yu-Bin Yan, Norm C. Duke, Mei Sun^*^**

*** Correspondence:** Corresponding Author: meisun@hku.hk

**Supplementary Table 1**. Null allele frequency as estimated by FREENA for each locus-population combination.

|  | **Rhst01** | **Rhst02** | **Rhst11** | **Rhst13** | **Rhst15** | **Rhst19** | **RS19** | **RS59** | **RS67** | **RM11** | **RM38** | **RM41** | **RM46** |
| --- | --- | --- | --- | --- | --- | --- | --- | --- | --- | --- | --- | --- | --- |
| ***R. apiculata*** |  |  |  |  |  |  |  |  |  |  |  |  |  |
| AUS .CTR | 0.055 | 0.001 | 0.033 | 0.000 | 0.068 | 0.000 | 0.000 | 0.045 | 0.000 | 0.000 | 0.000 | 0.000 | 0.001 |
| AUS .DTR | 0.182 | 0.001 | 0.000 | 0.000 | 0.000 | 0.000 | 0.000 | 0.000 | 0.025 | 0.000 | 0.001 | 0.001 | 0.001 |
| AUS .EMB | 0.071 | 0.001 | 0.000 | 0.089 | 0.071 | 0.031 | 0.134 | 0.000 | 0.000 | 0.000 | 0.001 | 0.001 | 0.001 |
| AUS .TRI | 0.001 | 0.000 | 0.117 | 0.000 | 0.000 | 0.000 | 0.000 | 0.112 | 0.000 | 0.000 | 0.001 | 0.001 | 0.001 |
| CHN .QLH | 0.114 | 0.093 | 0.001 | 0.000 | 0.050 | 0.001 | 0.098 | 0.150 | 0.038 | 0.000 | 0.000 | 0.000 | 0.001 |
| CHN .TLH | 0.222 | 0.000 | 0.033 | 0.005 | 0.000 | 0.001 | 0.000 | 0.000 | 0.000 | 0.000 | 0.000 | 0.033 | 0.001 |
| IDN .NSL | 0.000 | 0.000 | 0.000 | 0.019 | 0.000 | 0.000 | 0.000 | 0.030 | 0.043 | 0.000 | 0.001 | 0.126 | 0.001 |
| IDN .TRK | 0.000 | 0.283 | 0.143 | 0.000 | 0.147 | 0.000 | 0.000 | 0.000 | 0.000 | 0.000 | 0.001 | 0.110 | 0.001 |
| MCN .CHK | 0.170 | 0.022 | 0.000 | 0.000 | 0.129 | 0.000 | 0.102 | 0.000 | 0.000 | 0.000 | 0.001 | 0.001 | 0.001 |
| MCN .KSR | 0.000 | 0.178 | 0.000 | 0.000 | 0.052 | 0.001 | 0.001 | 0.000 | 0.000 | 0.000 | 0.001 | 0.001 | 0.001 |
| MCN .YAP | 0.325 | 0.097 | 0.130 | 0.021 | 0.127 | 0.028 | 0.000 | 0.200 | 0.000 | 0.000 | 0.039 | 0.000 | 0.001 |
| MLS .BLG | 0.000 | 0.005 | 0.000 | 0.000 | 0.000 | 0.000 | 0.000 | 0.000 | 0.000 | 0.000 | 0.000 | 0.106 | 0.001 |
| MLS .KPS | 0.245 | 0.000 | 0.000 | 0.000 | 0.000 | 0.090 | 0.000 | 0.081 | 0.030 | 0.000 | 0.001 | 0.069 | 0.001 |
| PHP .PNL | 0.240 | 0.216 | 0.000 | 0.042 | 0.000 | 0.000 | 0.001 | 0.000 | 0.000 | 0.000 | 0.001 | 0.150 | 0.001 |
| SRL .PBL | 0.337 | 0.000 | 0.001 | 0.129 | 0.135 | 0.084 | 0.000 | 0.271 | 0.000 | 0.000 | 0.000 | 0.133 | 0.001 |
| THL .KKN | 0.236 | 0.022 | 0.000 | 0.000 | 0.109 | 0.000 | 0.000 | 0.000 | 0.000 | 0.000 | 0.001 | 0.100 | 0.001 |
| THL .MNP | 0.241 | 0.297 | 0.094 | 0.262 | 0.000 | 0.135 | 0.142 | 0.155 | 0.163 | 0.000 | 0.000 | 0.000 | 0.001 |
| THL .PNB | 0.217 | 0.022 | 0.000 | 0.000 | 0.000 | 0.000 | 0.160 | 0.000 | 0.021 | 0.000 | 0.001 | 0.159 | 0.001 |
| THL .RNG | 0.247 | 0.000 | 0.000 | 0.000 | 0.001 | 0.000 | 0.029 | 0.018 | 0.014 | 0.000 | 0.000 | 0.136 | 0.001 |
| USA .GM | 0.001 | 0.192 | 0.000 | 0.000 | 0.000 | 0.000 | 0.000 | 0.172 | 0.000 | 0.000 | 0.000 | 0.001 | 0.000 |
| ***R. mucronata*** |  |  |  |  |  |  |  |  |  |  |  |  |  |
| AUS .DTR | 0.001 | 0.000 | 0.137 | 0.196 | 0.001 | 0.000 | 0.000 | 0.001 | 0.184 | 0.000 | 0.000 | 0.000 | 0.140 |
| AUS .TRI | 0.302 | 0.190 | 0.118 | 0.322 | 0.310 | 0.302 | 0.000 | 0.218 | 0.123 | 0.000 | 0.118 | 0.157 | 0.118 |
| IDN .NSL | 0.206 | 0.021 | 0.062 | 0.000 | 0.000 | 0.091 | 0.000 | 0.000 | 0.075 | 0.000 | 0.000 | 0.000 | 0.103 |
| IDN .TRK | 0.167 | 0.333 | 0.202 | 0.143 | 0.000 | 0.000 | 0.000 | 0.046 | 0.076 | 0.001 | 0.195 | 0.000 | 0.000 |
| KNY .MDC | 0.000 | 0.106 | 0.000 | 0.260 | 0.001 | 0.000 | 0.000 | 0.050 | 0.000 | 0.001 | 0.243 | 0.000 | 0.124 |
| MCN .KSR | 0.000 | 0.001 | 0.000 | 0.128 | 0.001 | 0.000 | 0.000 | 0.207 | 0.101 | 0.001 | 0.224 | 0.000 | 0.000 |
| MCN .YAP | 0.000 | 0.096 | 0.198 | 0.115 | 0.000 | 0.105 | 0.000 | 0.029 | 0.051 | 0.000 | 0.017 | 0.082 | 0.174 |
| MLS .KPS | 0.157 | 0.000 | 0.238 | 0.000 | 0.132 | 0.000 | 0.000 | 0.129 | 0.000 | 0.000 | 0.082 | 0.000 | 0.000 |
| PHP .PNL | 0.241 | 0.000 | 0.132 | 0.152 | 0.001 | 0.000 | 0.000 | 0.142 | 0.195 | 0.000 | 0.134 | 0.000 | 0.000 |
| SRL .PBL | 0.267 | 0.000 | 0.000 | 0.000 | 0.054 | 0.001 | 0.000 | 0.130 | 0.000 | 0.000 | 0.000 | 0.000 | 0.000 |
| SRL .RKW | 0.224 | 0.000 | 0.000 | 0.000 | 0.000 | 0.001 | 0.000 | 0.001 | 0.000 | 0.001 | 0.065 | 0.000 | 0.001 |
| THL .PNB | 0.269 | 0.000 | 0.098 | 0.000 | 0.066 | 0.000 | 0.000 | 0.000 | 0.000 | 0.000 | 0.000 | 0.000 | 0.126 |
| ***R. stylosa*** |  |  |  |  |  |  |  |  |  |  |  |  |  |
| AUS .CDR | 0.177 | 0.000 | 0.119 | 0.000 | 0.156 | 0.199 | 0.000 | 0.000 | 0.184 | 0.000 | 0.001 | 0.000 | 0.270 |
| AUS .CTR | 0.177 | 0.084 | 0.085 | 0.000 | 0.000 | 0.036 | 0.000 | 0.000 | 0.018 | 0.000 | 0.024 | 0.000 | 0.000 |
| AUS .DTR | 0.119 | 0.000 | 0.000 | 0.028 | 0.000 | 0.001 | 0.000 | 0.000 | 0.042 | 0.000 | 0.000 | 0.000 | 0.000 |
| AUS .EMB | 0.117 | 0.000 | 0.000 | 0.000 | 0.000 | 0.000 | 0.000 | 0.000 | 0.000 | 0.000 | 0.062 | 0.086 | 0.038 |
| AUS .MTB | 0.001 | 0.155 | 0.000 | 0.000 | 0.188 | 0.000 | 0.000 | 0.000 | 0.000 | 0.000 | 0.000 | 0.000 | 0.000 |
| AUS .SLB | 0.001 | 0.021 | 0.000 | 0.000 | 0.000 | 0.000 | 0.000 | 0.195 | 0.000 | 0.000 | 0.000 | 0.000 | 0.021 |
| AUS .TBC | 0.000 | 0.087 | 0.111 | 0.000 | 0.263 | 0.274 | 0.000 | 0.064 | 0.249 | 0.000 | 0.001 | 0.000 | 0.071 |
| AUS .TDR | 0.000 | 0.000 | 0.142 | 0.000 | 0.116 | 0.001 | 0.000 | 0.000 | 0.001 | 0.000 | 0.001 | 0.102 | 0.000 |
| AUS .TRI | 0.001 | 0.060 | 0.000 | 0.053 | 0.028 | 0.000 | 0.000 | 0.000 | 0.000 | 0.000 | 0.000 | 0.000 | 0.000 |
| CHN .DNZ | 0.001 | 0.267 | 0.000 | 0.000 | 0.190 | 0.000 | 0.000 | 0.000 | 0.000 | 0.001 | 0.001 | 0.001 | 0.001 |
| CHN .DZH | 0.085 | 0.000 | 0.001 | 0.000 | 0.189 | 0.001 | 0.000 | 0.000 | 0.001 | 0.000 | 0.000 | 0.001 | 0.000 |
| FJ .SVP | 0.182 | 0.141 | 0.000 | 0.000 | 0.001 | 0.001 | 0.000 | 0.117 | 0.001 | 0.000 | 0.001 | 0.001 | 0.182 |
| IDN .NSL | 0.085 | 0.000 | 0.127 | 0.000 | 0.029 | 0.031 | 0.000 | 0.135 | 0.000 | 0.000 | 0.063 | 0.001 | 0.000 |
| JPN.IRT | 0.137 | 0.000 | 0.146 | 0.000 | 0.042 | 0.079 | 0.000 | 0.094 | 0.000 | 0.000 | 0.001 | 0.001 | 0.000 |
| KRB .BTR | 0.001 | 0.000 | 0.137 | 0.000 | 0.001 | 0.001 | 0.000 | 0.157 | 0.001 | 0.001 | 0.227 | 0.001 | 0.001 |
| KRB .TRW | 0.247 | 0.157 | 0.001 | 0.000 | 0.001 | 0.001 | 0.000 | 0.001 | 0.137 | 0.000 | 0.130 | 0.060 | 0.001 |
| MCN .CHK | 0.227 | 0.157 | 0.121 | 0.000 | 0.211 | 0.099 | 0.000 | 0.001 | 0.000 | 0.000 | 0.001 | 0.000 | 0.195 |
| MCN .KSR | 0.210 | 0.000 | 0.197 | 0.000 | 0.000 | 0.000 | 0.000 | 0.000 | 0.000 | 0.001 | 0.213 | 0.000 | 0.000 |
| MCN .YAP | 0.000 | 0.080 | 0.165 | 0.000 | 0.000 | 0.001 | 0.000 | 0.161 | 0.001 | 0.000 | 0.000 | 0.106 | 0.064 |
| MLS .TKP | 0.000 | 0.000 | 0.000 | 0.000 | 0.000 | 0.000 | 0.000 | 0.000 | 0.000 | 0.000 | 0.000 | 0.001 | 0.000 |
| PHP .PNL | 0.316 | 0.197 | 0.001 | 0.000 | 0.254 | 0.001 | 0.000 | 0.209 | 0.125 | 0.000 | 0.176 | 0.001 | 0.001 |
| USA .GM | 0.001 | 0.000 | 0.001 | 0.000 | 0.000 | 0.000 | 0.000 | 0.127 | 0.001 | 0.000 | 0.001 | 0.001 | 0.001 |
| **Mean** | 0.126 | 0.066 | 0.057 | 0.036 | 0.058 | 0.030 | 0.012 | 0.064 | 0.035 | 0.000 | 0.038 | 0.032 | 0.031 |

**Supplementary Table 2.** Pairwise *F*_ST_ (Weir, 1996) estimates with FSTAT 2.9.3 (Goudet, 2001), both using the ENA correction (null alleles excluded; above diagonal) and without using it (null alleles included; below diagonal) for each pair of populations in *R. apiculata*, *R. mucronata*, and *R. stylosa*. Values in green denote non-significant genetic differentiation between populations; other values mean significant differentiation at the level *p* < 0.05 after Bonferroni correction.

1. *R. apiculata*

|  | **AUS.CTR** | **AUS.DTR** | **AUS. EMB** | **AUS. TRI** | **CHN.QLH** | **CHN.TLH** | **IDN. NSL** | **IDN. TRK** | **MCN.CHK** | **MCN.KSR** | **MCN.YAP** | **MLS.BLG** | **MLS.KPS** | **PHP. PNL** | **SRL. PBL** | **THL. KKN** | **THL.MNP** | **THL. PNB** | **THL. RNG** | **USA.GM** |
| --- | --- | --- | --- | --- | --- | --- | --- | --- | --- | --- | --- | --- | --- | --- | --- | --- | --- | --- | --- | --- |
| **AUS.CTR** | - | 0.63 | 0.11 | 0.59 | 0.64 | 0.64 | 0.50 | 0.50 | 0.62 | 0.67 | 0.45 | 0.51 | 0.54 | 0.46 | 0.56 | 0.52 | 0.40 | 0.48 | 0.49 | 0.63 |
| **AUS.DTR** | 0.64 | - | 0.58 | 0.02 | 0.74 | 0.72 | 0.58 | 0.63 | 0.64 | 0.66 | 0.54 | 0.56 | 0.61 | 0.58 | 0.64 | 0.63 | 0.52 | 0.56 | 0.54 | 0.64 |
| **AUS. EMB** | 0.11 | 0.59 | - | 0.52 | 0.56 | 0.58 | 0.39 | 0.36 | 0.56 | 0.62 | 0.37 | 0.43 | 0.47 | 0.35 | 0.51 | 0.48 | 0.30 | 0.42 | 0.42 | 0.56 |
| **AUS. TRI** | 0.60 | 0.01 | 0.54 | - | 0.72 | 0.70 | 0.53 | 0.59 | 0.61 | 0.63 | 0.49 | 0.52 | 0.58 | 0.53 | 0.62 | 0.61 | 0.48 | 0.53 | 0.51 | 0.61 |
| **CHN.QLH** | 0.65 | 0.75 | 0.58 | 0.73 | - | 0.42 | 0.39 | 0.36 | 0.69 | 0.72 | 0.52 | 0.44 | 0.51 | 0.33 | 0.59 | 0.58 | 0.38 | 0.50 | 0.53 | 0.71 |
| **CHN.TLH** | 0.65 | 0.73 | 0.60 | 0.71 | 0.43 | - | 0.35 | 0.39 | 0.65 | 0.69 | 0.47 | 0.40 | 0.44 | 0.30 | 0.52 | 0.52 | 0.34 | 0.45 | 0.46 | 0.67 |
| **IDN. NSL** | 0.51 | 0.60 | 0.40 | 0.54 | 0.40 | 0.36 | - | 0.09 | 0.50 | 0.56 | 0.30 | 0.23 | 0.29 | 0.18 | 0.41 | 0.38 | 0.15 | 0.29 | 0.33 | 0.49 |
| **IDN. TRK** | 0.51 | 0.64 | 0.38 | 0.59 | 0.37 | 0.39 | 0.08 | - | 0.53 | 0.60 | 0.31 | 0.21 | 0.27 | 0.14 | 0.39 | 0.38 | 0.13 | 0.28 | 0.31 | 0.53 |
| **MCN.CHK** | 0.63 | 0.65 | 0.57 | 0.62 | 0.71 | 0.66 | 0.51 | 0.53 | - | 0.27 | 0.24 | 0.51 | 0.54 | 0.50 | 0.57 | 0.54 | 0.43 | 0.49 | 0.45 | 0.25 |
| **MCN.KSR** | 0.67 | 0.66 | 0.63 | 0.64 | 0.74 | 0.70 | 0.56 | 0.60 | 0.28 | - | 0.35 | 0.53 | 0.57 | 0.55 | 0.62 | 0.57 | 0.50 | 0.51 | 0.49 | 0.01 |
| **MCN.YAP** | 0.46 | 0.55 | 0.38 | 0.49 | 0.54 | 0.48 | 0.31 | 0.31 | 0.24 | 0.36 | - | 0.37 | 0.40 | 0.28 | 0.45 | 0.40 | 0.25 | 0.36 | 0.33 | 0.30 |
| **MLS.BLG** | 0.52 | 0.58 | 0.45 | 0.53 | 0.46 | 0.41 | 0.23 | 0.21 | 0.52 | 0.54 | 0.38 | - | 0.07 | 0.25 | 0.18 | 0.17 | 0.14 | 0.10 | 0.17 | 0.50 |
| **MLS.KPS** | 0.55 | 0.63 | 0.48 | 0.59 | 0.53 | 0.45 | 0.30 | 0.27 | 0.56 | 0.58 | 0.41 | 0.08 | - | 0.29 | 0.13 | 0.14 | 0.20 | 0.05 | 0.11 | 0.54 |
| **PHP. PNL** | 0.47 | 0.59 | 0.36 | 0.54 | 0.34 | 0.31 | 0.18 | 0.14 | 0.51 | 0.56 | 0.30 | 0.26 | 0.30 | - | 0.37 | 0.34 | 0.17 | 0.27 | 0.29 | 0.50 |
| **SRL. PBL** | 0.58 | 0.66 | 0.53 | 0.64 | 0.61 | 0.54 | 0.42 | 0.40 | 0.60 | 0.63 | 0.48 | 0.19 | 0.14 | 0.40 | - | 0.09 | 0.26 | 0.08 | 0.12 | 0.59 |
| **THL. KKN** | 0.53 | 0.64 | 0.49 | 0.61 | 0.60 | 0.53 | 0.38 | 0.37 | 0.55 | 0.57 | 0.41 | 0.17 | 0.14 | 0.35 | 0.10 | - | 0.23 | 0.04 | 0.10 | 0.54 |
| **THL.MNP** | 0.41 | 0.53 | 0.31 | 0.47 | 0.39 | 0.33 | 0.14 | 0.12 | 0.43 | 0.50 | 0.27 | 0.14 | 0.22 | 0.19 | 0.29 | 0.24 | - | 0.18 | 0.20 | 0.44 |
| **THL. PNB** | 0.48 | 0.56 | 0.42 | 0.52 | 0.51 | 0.45 | 0.29 | 0.28 | 0.50 | 0.51 | 0.37 | 0.11 | 0.05 | 0.28 | 0.09 | 0.03 | 0.19 | - | 0.05 | 0.48 |
| **THL. RNG** | 0.50 | 0.55 | 0.42 | 0.51 | 0.54 | 0.47 | 0.33 | 0.31 | 0.46 | 0.48 | 0.35 | 0.18 | 0.11 | 0.30 | 0.14 | 0.10 | 0.21 | 0.05 | - | 0.45 |
| **USA.GM** | 0.64 | 0.65 | 0.57 | 0.62 | 0.72 | 0.68 | 0.50 | 0.53 | 0.26 | 0.01 | 0.31 | 0.51 | 0.55 | 0.51 | 0.61 | 0.54 | 0.44 | 0.48 | 0.46 | - |

1. *R. mucronata*

|  | **AUS.DTR** | **AUS.TRI** | **IDN.NSL** | **IDN.TRK** | **KNY.MDC** | **MCN.KSR** | **MCN.YAP** | **MLS.KPS** | **PHP.PNL** | **SRL.PBL** | **SRL.RKW** | **THL.PNB** |
| --- | --- | --- | --- | --- | --- | --- | --- | --- | --- | --- | --- | --- |
| **AUS.DTR** | - | 0.22 | 0.58 | 0.56 | 0.65 | 0.60 | 0.52 | 0.59 | 0.55 | 0.64 | 0.70 | 0.66 |
| **AUS.TRI** | 0.19 | - | 0.31 | 0.24 | 0.34 | 0.34 | 0.25 | 0.36 | 0.31 | 0.37 | 0.42 | 0.47 |
| **IDN.NSL** | 0.61 | 0.34 | - | 0.07 | 0.47 | 0.29 | 0.32 | 0.31 | 0.33 | 0.48 | 0.48 | 0.36 |
| **IDN.TRK** | 0.60 | 0.27 | 0.08 | - | 0.47 | 0.24 | 0.27 | 0.39 | 0.36 | 0.51 | 0.54 | 0.49 |
| **KNY.MDC** | 0.68 | 0.36 | 0.49 | 0.50 | - | 0.54 | 0.42 | 0.44 | 0.41 | 0.42 | 0.42 | 0.52 |
| **MCN.KSR** | 0.64 | 0.35 | 0.31 | 0.24 | 0.56 | - | 0.34 | 0.49 | 0.43 | 0.58 | 0.62 | 0.57 |
| **MCN.YAP** | 0.56 | 0.29 | 0.33 | 0.29 | 0.44 | 0.35 | - | 0.31 | 0.23 | 0.43 | 0.44 | 0.44 |
| **MLS.KPS** | 0.62 | 0.39 | 0.32 | 0.41 | 0.48 | 0.51 | 0.34 | - | 0.15 | 0.46 | 0.45 | 0.08 |
| **PHP.PNL** | 0.57 | 0.34 | 0.35 | 0.39 | 0.44 | 0.45 | 0.25 | 0.18 | - | 0.42 | 0.40 | 0.28 |
| **SRL.PBL** | 0.67 | 0.40 | 0.49 | 0.54 | 0.45 | 0.60 | 0.45 | 0.49 | 0.44 | - | 0.25 | 0.52 |
| **SRL.RKW** | 0.72 | 0.43 | 0.49 | 0.56 | 0.45 | 0.64 | 0.45 | 0.48 | 0.41 | 0.27 | - | 0.51 |
| **THL.PNB** | 0.69 | 0.49 | 0.38 | 0.52 | 0.55 | 0.59 | 0.46 | 0.07 | 0.29 | 0.56 | 0.55 | - |

1. *R. stylosa*

|  | **AUS.CDR** | **AUS.CTR** | **AUS.DTR** | **AUS.EMB** | **AUS.MTB** | **AUS.SLB** | **AUS.TBC** | **AUS.TDR** | **AUS.TRI** | **CHN.DNZ** | **CHN.DZH** | **FJ. SVP** | **IDN.NSL** | **JPN. IRT** | **KRB.BTR** | **KRB.TRW** | **MCN.CHK** | **MCN.KSR** | **MCN.YAP** | **MLS.TKP** | **PHP.PNL** | **USA.GM** |
| --- | --- | --- | --- | --- | --- | --- | --- | --- | --- | --- | --- | --- | --- | --- | --- | --- | --- | --- | --- | --- | --- | --- |
| **AUS.CDR** | - | 0.25 | 0.18 | 0.30 | 0.07 | 0.10 | 0.08 | 0.11 | 0.10 | 0.60 | 0.62 | 0.34 | 0.45 | 0.55 | 0.60 | 0.57 | 0.38 | 0.46 | 0.49 | 0.59 | 0.51 | 0.61 |
| **AUS.CTR** | 0.28 | - | 0.27 | 0.07 | 0.26 | 0.21 | 0.30 | 0.32 | 0.26 | 0.39 | 0.43 | 0.29 | 0.28 | 0.40 | 0.44 | 0.41 | 0.28 | 0.33 | 0.32 | 0.42 | 0.31 | 0.45 |
| **AUS.DTR** | 0.20 | 0.27 | - | 0.30 | 0.13 | 0.18 | 0.17 | 0.16 | 0.09 | 0.58 | 0.61 | 0.38 | 0.44 | 0.54 | 0.54 | 0.51 | 0.39 | 0.43 | 0.45 | 0.59 | 0.49 | 0.57 |
| **AUS.EMB** | 0.33 | 0.07 | 0.30 | - | 0.29 | 0.25 | 0.33 | 0.38 | 0.29 | 0.46 | 0.51 | 0.36 | 0.33 | 0.45 | 0.52 | 0.49 | 0.35 | 0.39 | 0.38 | 0.49 | 0.36 | 0.54 |
| **AUS.MTB** | 0.07 | 0.26 | 0.12 | 0.30 | - | 0.10 | 0.07 | 0.03 | 0.06 | 0.64 | 0.66 | 0.36 | 0.49 | 0.57 | 0.61 | 0.58 | 0.42 | 0.48 | 0.49 | 0.64 | 0.53 | 0.63 |
| **AUS.SLB** | 0.10 | 0.21 | 0.19 | 0.25 | 0.11 | - | 0.06 | 0.18 | 0.12 | 0.58 | 0.61 | 0.43 | 0.39 | 0.49 | 0.62 | 0.58 | 0.35 | 0.41 | 0.44 | 0.57 | 0.42 | 0.65 |
| **AUS.TBC** | 0.09 | 0.31 | 0.17 | 0.35 | 0.06 | 0.06 | - | 0.08 | 0.10 | 0.59 | 0.61 | 0.40 | 0.47 | 0.52 | 0.60 | 0.57 | 0.38 | 0.46 | 0.48 | 0.59 | 0.50 | 0.60 |
| **AUS.TDR** | 0.10 | 0.33 | 0.17 | 0.39 | 0.03 | 0.18 | 0.06 | - | 0.11 | 0.69 | 0.70 | 0.44 | 0.54 | 0.62 | 0.67 | 0.64 | 0.48 | 0.55 | 0.55 | 0.68 | 0.59 | 0.67 |
| **AUS.TRI** | 0.11 | 0.26 | 0.08 | 0.29 | 0.06 | 0.12 | 0.09 | 0.11 | - | 0.62 | 0.65 | 0.36 | 0.47 | 0.56 | 0.59 | 0.56 | 0.38 | 0.44 | 0.48 | 0.62 | 0.51 | 0.63 |
| **CHN.DNZ** | 0.63 | 0.39 | 0.60 | 0.47 | 0.65 | 0.58 | 0.61 | 0.70 | 0.63 | - | 0.22 | 0.70 | 0.28 | 0.41 | 0.70 | 0.68 | 0.38 | 0.50 | 0.55 | 0.30 | 0.24 | 0.75 |
| **CHN.DZH** | 0.65 | 0.44 | 0.63 | 0.52 | 0.68 | 0.62 | 0.63 | 0.72 | 0.66 | 0.24 | - | 0.71 | 0.32 | 0.43 | 0.69 | 0.68 | 0.38 | 0.52 | 0.56 | 0.36 | 0.26 | 0.73 |
| **FJ. SVP** | 0.39 | 0.31 | 0.42 | 0.38 | 0.39 | 0.46 | 0.41 | 0.47 | 0.40 | 0.71 | 0.73 | - | 0.53 | 0.67 | 0.70 | 0.65 | 0.47 | 0.56 | 0.61 | 0.70 | 0.60 | 0.74 |
| **IDN.NSL** | 0.48 | 0.29 | 0.46 | 0.34 | 0.50 | 0.39 | 0.48 | 0.56 | 0.48 | 0.30 | 0.34 | 0.55 | - | 0.28 | 0.49 | 0.50 | 0.28 | 0.35 | 0.34 | 0.26 | 0.14 | 0.56 |
| **JPN. IRT** | 0.58 | 0.41 | 0.55 | 0.47 | 0.59 | 0.51 | 0.55 | 0.64 | 0.57 | 0.45 | 0.47 | 0.70 | 0.30 | - | 0.60 | 0.63 | 0.38 | 0.41 | 0.39 | 0.34 | 0.14 | 0.70 |
| **KRB.BTR** | 0.62 | 0.45 | 0.55 | 0.53 | 0.61 | 0.63 | 0.60 | 0.67 | 0.59 | 0.71 | 0.71 | 0.72 | 0.50 | 0.62 | - | 0.42 | 0.40 | 0.27 | 0.52 | 0.65 | 0.55 | 0.61 |
| **KRB.TRW** | 0.61 | 0.43 | 0.55 | 0.51 | 0.60 | 0.60 | 0.59 | 0.66 | 0.59 | 0.70 | 0.70 | 0.69 | 0.52 | 0.65 | 0.42 | - | 0.33 | 0.37 | 0.57 | 0.65 | 0.55 | 0.69 |
| **MCN.CHK** | 0.43 | 0.30 | 0.40 | 0.37 | 0.43 | 0.36 | 0.40 | 0.49 | 0.39 | 0.40 | 0.41 | 0.48 | 0.29 | 0.41 | 0.40 | 0.36 | - | 0.20 | 0.38 | 0.39 | 0.31 | 0.52 |
| **MCN.KSR** | 0.49 | 0.33 | 0.44 | 0.39 | 0.47 | 0.41 | 0.46 | 0.56 | 0.44 | 0.50 | 0.53 | 0.57 | 0.35 | 0.44 | 0.28 | 0.39 | 0.20 | - | 0.40 | 0.45 | 0.36 | 0.55 |
| **MCN.YAP** | 0.52 | 0.33 | 0.47 | 0.39 | 0.52 | 0.46 | 0.51 | 0.58 | 0.50 | 0.56 | 0.58 | 0.64 | 0.36 | 0.41 | 0.53 | 0.59 | 0.41 | 0.41 | - | 0.52 | 0.38 | 0.58 |
| **MLS.TKP** | 0.61 | 0.42 | 0.59 | 0.48 | 0.64 | 0.57 | 0.60 | 0.69 | 0.62 | 0.31 | 0.38 | 0.70 | 0.26 | 0.36 | 0.65 | 0.67 | 0.40 | 0.44 | 0.53 | - | 0.25 | 0.74 |
| **PHP.PNL** | 0.53 | 0.33 | 0.50 | 0.37 | 0.53 | 0.42 | 0.51 | 0.60 | 0.51 | 0.26 | 0.29 | 0.61 | 0.17 | 0.12 | 0.56 | 0.57 | 0.33 | 0.37 | 0.37 | 0.25 | - | 0.63 |
| **USA.GM** | 0.63 | 0.45 | 0.58 | 0.54 | 0.64 | 0.66 | 0.61 | 0.68 | 0.64 | 0.75 | 0.74 | 0.76 | 0.57 | 0.72 | 0.62 | 0.71 | 0.53 | 0.55 | 0.60 | 0.75 | 0.63 | - |

**Supplementary Table 3.** Distribution and frequencies of private alleles in populations of *R. apiculate*, *R. mucronata*, and *R. stylosa*.

1. Distribution of private alleles.

| **Species** | **Population** | **No. of private alleles** |
| --- | --- | --- |
| ***R. apiculata*** |  |  |
|  | AUS.CTR | 4 |
|  | AUS.EMB | 1 |
|  | MCN.YAP | 3 |
|  | PHP.PNL | 4 |
|  | SRL.PBL | 1 |
|  | THL.MNP | 7 |
|  | THL.PNB | 2 |
|  | THL.RNG | 1 |
|  | USA.GM | 2 |
| ***R. mucronata*** |  |  |
|  | AUS.DTR | 1 |
|  | AUS.TRI | 5 |
|  | IDN.NSL | 5 |
|  | IDN.TRK | 3 |
|  | KNY.MDC | 6 |
|  | MCN.KSR | 1 |
|  | MCN.YAP | 7 |
|  | MLS.KPS | 3 |
|  | PHP.PNL | 6 |
|  | SRL.PBL | 3 |
|  | SRL.RKW | 1 |
| ***R. stylosa*** |  |  |
|  | AUS.CDR | 2 |
|  | AUS.CTR | 5 |
|  | AUS.DTR | 1 |
|  | AUS.EMB | 2 |
|  | AUS.MTB | 1 |
|  | AUS.TDR | 1 |
|  | FJ.SVP | 1 |
|  | IDN.NSL | 1 |
|  | JPN.IRT | 1 |
|  | KRB.TRW | 2 |
|  | MCN.CHK | 2 |
|  | MCN.KSR | 2 |
|  | MCN.YAP | 3 |
|  | MLS.TKP | 1 |

1. Frequencies of private alleles.

| **Species** | **Population** | **Locus** | **Allele** | **Frequency** |
| --- | --- | --- | --- | --- |
| ***R. apiculata*** |  |  |  |  |
|  | AUS.CTR | Rhst11 | 137 | 0.071 |
|  | AUS.CTR | RS59 | 120 | 0.024 |
|  | AUS.CTR | RM38 | 202 | 0.024 |
|  | AUS.CTR | RM41 | 173 | 0.024 |
|  | AUS.EMB | RS19 | 142 | 0.045 |
|  | MCN.YAP | Rhst02 | 165 | 0.026 |
|  | MCN.YAP | Rhst15 | 196 | 0.026 |
|  | MCN.YAP | RS67 | 148 | 0.026 |
|  | PHP.PNL | Rhst11 | 133 | 0.029 |
|  | PHP.PNL | Rhst11 | 135 | 0.206 |
|  | PHP.PNL | Rhst13 | 146 | 0.088 |
|  | PHP.PNL | RM41 | 177 | 0.118 |
|  | SRL.PBL | Rhst01 | 229 | 0.023 |
|  | THL.MNP | Rhst01 | 271 | 0.067 |
|  | THL.MNP | Rhst01 | 275 | 0.167 |
|  | THL.MNP | Rhst01 | 277 | 0.233 |
|  | THL.MNP | Rhst01 | 281 | 0.067 |
|  | THL.MNP | Rhst19 | 114 | 0.063 |
|  | THL.MNP | RS19 | 162 | 0.031 |
|  | THL.MNP | RS67 | 136 | 0.469 |
|  | THL.PNB | Rhst11 | 155 | 0.019 |
|  | THL.PNB | Rhst13 | 132 | 0.019 |
|  | THL.RNG | Rhst01 | 228 | 0.021 |
|  | USA.GM | RM38 | 216 | 0.050 |
|  | USA.GM | RM46 | 182 | 0.050 |
| ***R. mucronata*** |  |  |  |  |
|  | AUS.DTR | Rhst11 | 155 | 0.042 |
|  | AUS.TRI | Rhst02 | 175 | 0.071 |
|  | AUS.TRI | Rhst15 | 190 | 0.143 |
|  | AUS.TRI | Rhst15 | 212 | 0.071 |
|  | AUS.TRI | RS67 | 142 | 0.286 |
|  | AUS.TRI | RM41 | 171 | 0.071 |
|  | IDN.NSL | Rhst02 | 163 | 0.115 |
|  | IDN.NSL | Rhst02 | 181 | 0.038 |
|  | IDN.NSL | Rhst11 | 137 | 0.038 |
|  | IDN.NSL | Rhst11 | 157 | 0.038 |
|  | IDN.NSL | Rhst19 | 114 | 0.038 |
|  | IDN.TRK | Rhst13 | 126 | 0.167 |
|  | IDN.TRK | Rhst13 | 140 | 0.083 |
|  | IDN.TRK | Rhst15 | 210 | 0.083 |
|  | KNY.MDC | Rhst11 | 143 | 0.077 |
|  | KNY.MDC | Rhst11 | 165 | 0.115 |
|  | KNY.MDC | Rhst11 | 167 | 0.231 |
|  | KNY.MDC | Rhst13 | 166 | 0.192 |
|  | KNY.MDC | RS67 | 164 | 0.077 |
|  | KNY.MDC | RM46 | 184 | 0.038 |
|  | MCN.KSR | Rhst01 | 226 | 0.063 |
|  | MCN.YAP | Rhst13 | 176 | 0.033 |
|  | MCN.YAP | Rhst15 | 202 | 0.100 |
|  | MCN.YAP | Rhst15 | 208 | 0.033 |
|  | MCN.YAP | Rhst19 | 106 | 0.033 |
|  | MCN.YAP | RS67 | 156 | 0.567 |
|  | MCN.YAP | RS67 | 160 | 0.033 |
|  | MCN.YAP | RM11 | 216 | 0.033 |
|  | MLS.KPS | Rhst13 | 150 | 0.028 |
|  | MLS.KPS | RS19 | 146 | 0.028 |
|  | MLS.KPS | RS67 | 144 | 0.083 |
|  | PHP.PNL | Rhst13 | 180 | 0.023 |
|  | PHP.PNL | Rhst19 | 102 | 0.023 |
|  | PHP.PNL | RS19 | 170 | 0.023 |
|  | PHP.PNL | RS19 | 172 | 0.023 |
|  | PHP.PNL | RS59 | 148 | 0.023 |
|  | PHP.PNL | RM38 | 218 | 0.136 |
|  | SRL.PBL | RS59 | 120 | 0.792 |
|  | SRL.PBL | RM11 | 196 | 0.042 |
|  | SRL.PBL | RM11 | 204 | 0.042 |
|  | SRL.RKW | Rhst15 | 206 | 0.063 |
| ***R. stylosa*** |  |  |  |  |
|  | AUS.CDR | Rhst11 | 155 | 0.024 |
|  | AUS.CDR | RS59 | 144 | 0.024 |
|  | AUS.CTR | Rhst13 | 154 | 0.022 |
|  | AUS.CTR | Rhst13 | 166 | 0.022 |
|  | AUS.CTR | RS59 | 134 | 0.043 |
|  | AUS.CTR | RS67 | 158 | 0.022 |
|  | AUS.CTR | RM38 | 194 | 0.022 |
|  | AUS.DTR | RM38 | 230 | 0.042 |
|  | AUS.EMB | Rhst11 | 137 | 0.100 |
|  | AUS.EMB | RM41 | 151 | 0.063 |
|  | AUS.MTB | Rhst19 | 100 | 0.031 |
|  | AUS.TDR | Rhst13 | 150 | 0.020 |
|  | FJ.SVP | RS59 | 132 | 0.038 |
|  | IDN.NSL | RM38 | 232 | 0.118 |
|  | JPN.IRT | Rhst19 | 116 | 0.083 |
|  | KRB.TRW | Rhst13 | 156 | 0.053 |
|  | KRB.TRW | RM38 | 236 | 0.079 |
|  | MCN.CHK | Rhst01 | 231 | 0.050 |
|  | MCN.CHK | RM46 | 184 | 0.025 |
|  | MCN.KSR | RM38 | 190 | 0.042 |
|  | MCN.KSR | RM41 | 177 | 0.042 |
|  | MCN.YAP | Rhst13 | 168 | 0.025 |
|  | MCN.YAP | Rhst13 | 170 | 0.050 |
|  | MCN.YAP | RM11 | 204 | 0.025 |
|  | MLS.TKP | RM38 | 206 | 0.029 |

**Supplementary Table 4.** Migration rate between populations inferred from BayesAss analysis. For all the values, the direction of migration is from the corresponding population in the first column to the corresponding population in the first row. Highlighted values in green are population pairs with high levels of asymmetry in gene flow.

1. *R. apiculata*

|  | **AUS. CTR** | **AUS. DTR** | **AUS. EMB** | **AUS. TRI** | **CHN. QLH** | **CHN. TLH** | **IDN. NSL** | **IDN. TRK** | **MCN. CHK** | **MCN.KSR** | **MCN. YAP** | **MLS. BLG** | **MLS. KPS** | **PHP. PNL** | **SRL. PBL** | **THL. KKN** | **THL. MNP** | **THL. PNB** | **THL. RNG** | **USA. GM** |
| --- | --- | --- | --- | --- | --- | --- | --- | --- | --- | --- | --- | --- | --- | --- | --- | --- | --- | --- | --- | --- |
| **AUS. CTR** | - | 0.0078 | 0.0077 | 0.0078 | 0.0077 | 0.0078 | 0.0078 | 0.0077 | 0.0077 | 0.0078 | 0.0078 | 0.0077 | 0.0077 | 0.0077 | 0.0078 | 0.0077 | 0.0077 | 0.0076 | 0.0077 | 0.0078 |
| **AUS. DTR** | 0.0069 | - | 0.0069 | 0.0069 | 0.0069 | 0.0070 | 0.0070 | 0.0070 | 0.0068 | 0.0069 | 0.0068 | 0.0069 | 0.0069 | 0.0070 | 0.0069 | 0.0069 | 0.0069 | 0.0069 | 0.0069 | 0.0069 |
| **AUS. EMB** | 0.1285 | 0.0108 | - | 0.0107 | 0.0107 | 0.0106 | 0.0108 | 0.0107 | 0.0108 | 0.0107 | 0.0107 | 0.0108 | 0.0107 | 0.0107 | 0.0108 | 0.0108 | 0.0107 | 0.0108 | 0.0108 | 0.0109 |
| **AUS. TRI** | 0.0103 | 0.1389 | 0.0102 | - | 0.0101 | 0.0101 | 0.0102 | 0.0102 | 0.0103 | 0.0103 | 0.0102 | 0.0102 | 0.0102 | 0.0103 | 0.0101 | 0.0103 | 0.0100 | 0.0103 | 0.0102 | 0.0103 |
| **CHN. QLH** | 0.0072 | 0.0070 | 0.0071 | 0.0070 | - | 0.0072 | 0.0071 | 0.0070 | 0.0071 | 0.0071 | 0.0072 | 0.0071 | 0.0071 | 0.0071 | 0.0071 | 0.0071 | 0.0070 | 0.0070 | 0.0071 | 0.0070 |
| **CHN. TLH** | 0.0071 | 0.0070 | 0.0071 | 0.0070 | 0.0080 | - | 0.0071 | 0.0070 | 0.0071 | 0.0071 | 0.0071 | 0.0071 | 0.0071 | 0.0072 | 0.0071 | 0.0071 | 0.0071 | 0.0071 | 0.0070 | 0.0071 |
| **IDN. NSL** | 0.0106 | 0.0105 | 0.0106 | 0.0107 | 0.0107 | 0.0112 | - | 0.0106 | 0.0106 | 0.0106 | 0.0107 | 0.0145 | 0.0105 | 0.0119 | 0.0109 | 0.0107 | 0.0108 | 0.0107 | 0.0105 | 0.0107 |
| **IDN. TRK** | 0.0126 | 0.0127 | 0.0127 | 0.0129 | 0.0127 | 0.0127 | 0.0898 | - | 0.0128 | 0.0128 | 0.0127 | 0.0128 | 0.0126 | 0.0128 | 0.0130 | 0.0127 | 0.0128 | 0.0128 | 0.0129 | 0.0127 |
| **MCN. CHK** | 0.0092 | 0.0094 | 0.0093 | 0.0092 | 0.0092 | 0.0093 | 0.0093 | 0.0094 | - | 0.0180 | 0.0095 | 0.0094 | 0.0093 | 0.0094 | 0.0092 | 0.0092 | 0.0094 | 0.0094 | 0.0094 | 0.0093 |
| **MCN.KSR** | 0.0081 | 0.0083 | 0.0082 | 0.0083 | 0.0081 | 0.0083 | 0.0083 | 0.0082 | 0.0090 | - | 0.0084 | 0.0083 | 0.0082 | 0.0082 | 0.0082 | 0.0082 | 0.0081 | 0.0082 | 0.0082 | 0.0082 |
| **MCN. YAP** | 0.0084 | 0.0085 | 0.0085 | 0.0084 | 0.0084 | 0.0084 | 0.0083 | 0.0084 | 0.0322 | 0.0112 | - | 0.0084 | 0.0083 | 0.0084 | 0.0085 | 0.0084 | 0.0085 | 0.0084 | 0.0085 | 0.0083 |
| **MLS. BLG** | 0.0083 | 0.0084 | 0.0084 | 0.0084 | 0.0083 | 0.0084 | 0.0084 | 0.0083 | 0.0084 | 0.0085 | 0.0083 | - | 0.0085 | 0.0083 | 0.1186 | 0.0084 | 0.0084 | 0.0575 | 0.0084 | 0.0083 |
| **MLS. KPS** | 0.0090 | 0.0091 | 0.0089 | 0.0090 | 0.0090 | 0.0090 | 0.0091 | 0.0091 | 0.0090 | 0.0091 | 0.0090 | 0.0092 | - | 0.0091 | 0.0146 | 0.0090 | 0.0090 | 0.1556 | 0.0090 | 0.0091 |
| **PHP. PNL** | 0.0088 | 0.0087 | 0.0088 | 0.0088 | 0.0108 | 0.0155 | 0.0109 | 0.0087 | 0.0087 | 0.0087 | 0.0088 | 0.0093 | 0.0088 | - | 0.0088 | 0.0088 | 0.0089 | 0.0088 | 0.0087 | 0.0087 |
| **SRL. PBL** | 0.0077 | 0.0077 | 0.0077 | 0.0077 | 0.0077 | 0.0077 | 0.0077 | 0.0077 | 0.0078 | 0.0076 | 0.0077 | 0.0080 | 0.0076 | 0.0076 | - | 0.0077 | 0.0076 | 0.0288 | 0.0077 | 0.0077 |
| **THL. KKN** | 0.0091 | 0.0093 | 0.0091 | 0.0091 | 0.0091 | 0.0091 | 0.0093 | 0.0091 | 0.0092 | 0.0091 | 0.0090 | 0.0091 | 0.0092 | 0.0092 | 0.0187 | - | 0.0092 | 0.1494 | 0.0092 | 0.0091 |
| **THL. MNP** | 0.0092 | 0.0093 | 0.0093 | 0.0092 | 0.0093 | 0.0092 | 0.0659 | 0.0092 | 0.0093 | 0.0092 | 0.0092 | 0.0151 | 0.0093 | 0.0093 | 0.0091 | 0.0093 | - | 0.0092 | 0.0091 | 0.0093 |
| **THL. PNB** | 0.0069 | 0.0069 | 0.0067 | 0.0069 | 0.0069 | 0.0069 | 0.0073 | 0.0069 | 0.0069 | 0.0069 | 0.0069 | 0.0076 | 0.0068 | 0.0069 | 0.0207 | 0.0070 | 0.0237 | - | 0.0068 | 0.0068 |
| **THL. RNG** | 0.0080 | 0.0081 | 0.0080 | 0.0080 | 0.0080 | 0.0080 | 0.0080 | 0.0080 | 0.0080 | 0.0080 | 0.0081 | 0.0080 | 0.0080 | 0.0080 | 0.0085 | 0.0080 | 0.0160 | 0.1721 | - | 0.0080 |
| **USA. GM** | 0.0111 | 0.0112 | 0.0111 | 0.0110 | 0.0111 | 0.0113 | 0.0111 | 0.0111 | 0.0113 | 0.1219 | 0.0110 | 0.0110 | 0.0110 | 0.0111 | 0.0111 | 0.0109 | 0.0111 | 0.0112 | 0.0110 | - |

1. *R. mucronata*

|  | **AUS.DTR** | **AUS.TRI** | **IDN.NSL** | **IDN.TRK** | **KNY.MDC** | **MCN.KSR** | **MCN.YAP** | **MLS.KPS** | **PHP.PNL** | **SRL.PBL** | **SRL.RKW** | **THL.PNB** |
| --- | --- | --- | --- | --- | --- | --- | --- | --- | --- | --- | --- | --- |
| **AUS.DTR** | - | 0.0089 | 0.0089 | 0.0088 | 0.0088 | 0.0368 | 0.0088 | 0.0088 | 0.0088 | 0.0088 | 0.0088 | 0.0088 |
| **AUS.TRI** | 0.0866 | - | 0.0174 | 0.0176 | 0.0523 | 0.0351 | 0.0176 | 0.0175 | 0.0175 | 0.0175 | 0.0175 | 0.0176 |
| **IDN.NSL** | 0.0128 | 0.0131 | - | 0.0130 | 0.0129 | 0.0389 | 0.0170 | 0.0131 | 0.0186 | 0.0136 | 0.0131 | 0.0137 |
| **IDN.TRK** | 0.0186 | 0.0188 | 0.1057 | - | 0.0188 | 0.0394 | 0.0187 | 0.0184 | 0.0188 | 0.0186 | 0.0185 | 0.0186 |
| **KNY.MDC** | 0.0127 | 0.0125 | 0.0126 | 0.0126 | - | 0.0126 | 0.0125 | 0.0126 | 0.0127 | 0.0124 | 0.0126 | 0.0127 |
| **MCN.KSR** | 0.0169 | 0.0168 | 0.1179 | 0.0168 | 0.0169 | - | 0.0333 | 0.0170 | 0.0169 | 0.0169 | 0.0168 | 0.0170 |
| **MCN.YAP** | 0.0115 | 0.0117 | 0.0117 | 0.0118 | 0.0116 | 0.0206 | - | 0.0119 | 0.0135 | 0.0116 | 0.0119 | 0.0117 |
| **MLS.KPS** | 0.0117 | 0.0117 | 0.0116 | 0.0115 | 0.0116 | 0.0116 | 0.0117 | - | 0.0116 | 0.0113 | 0.0116 | 0.2052 |
| **PHP.PNL** | 0.0094 | 0.0092 | 0.0094 | 0.0093 | 0.0092 | 0.0285 | 0.0134 | 0.0092 | - | 0.0093 | 0.0092 | 0.0222 |
| **SRL.PBL** | 0.0133 | 0.0129 | 0.0131 | 0.0132 | 0.0131 | 0.0130 | 0.0132 | 0.0130 | 0.0130 | - | 0.0130 | 0.0130 |
| **SRL.RKW** | 0.0173 | 0.0173 | 0.0172 | 0.0172 | 0.0171 | 0.0171 | 0.0173 | 0.0172 | 0.0173 | 0.1425 | - | 0.0173 |
| **THL.PNB** | 0.0093 | 0.0094 | 0.0092 | 0.0094 | 0.0092 | 0.0093 | 0.0093 | 0.0093 | 0.0123 | 0.0095 | 0.0094 | - |

1. *R. stylosa*

|  | **AUS. CDR** | **AUS. CTR** | **AUS. DTR** | **AUS. EMB** | **AUS. MTB** | **AUS. SLB** | **AUS. TBC** | **AUS. TDR** | **AUS. TRI** | **CHN. DNZ** | **CHN. DZH** | **FJ. SVP** | **IDN. NSL** | **JPN. IRT** | **KRB. BTR** | **KRB. TRW** | **MCN. CHK** | **MCN. KSR** | **MCN. YAP** | **MLS. TKP** | **PHP. PNL** | **USA. GM** |
| --- | --- | --- | --- | --- | --- | --- | --- | --- | --- | --- | --- | --- | --- | --- | --- | --- | --- | --- | --- | --- | --- | --- |
|  | **1** | **2** | **3** | **4** | **5** | **6** | **7** | **8** | **9** | **10** | **11** | **12** | **13** | **14** | **15** | **16** | **17** | **18** | **19** | **20** | **21** | **22** |
| **AUS. CDR** | - | 0.0079 | 0.0084 | 0.0080 | 0.0079 | 0.0079 | 0.0235 | 0.1513 | 0.0078 | 0.0079 | 0.0078 | 0.0078 | 0.0079 | 0.0078 | 0.0078 | 0.0078 | 0.0079 | 0.0079 | 0.0079 | 0.0079 | 0.0079 | 0.0080 |
| **AUS. CTR** | 0.0072 | - | 0.0073 | 0.0072 | 0.0072 | 0.0069 | 0.0072 | 0.0099 | 0.0071 | 0.0072 | 0.0071 | 0.0090 | 0.0070 | 0.0070 | 0.0071 | 0.0071 | 0.0071 | 0.0072 | 0.0074 | 0.0071 | 0.0071 | 0.0071 |
| **AUS. DTR** | 0.0071 | 0.0072 | - | 0.0071 | 0.0070 | 0.0072 | 0.0071 | 0.0328 | 0.0070 | 0.0070 | 0.0071 | 0.0071 | 0.0070 | 0.0070 | 0.0071 | 0.0071 | 0.0071 | 0.0071 | 0.0072 | 0.0071 | 0.0071 | 0.0070 |
| **AUS. EMB** | 0.0090 | 0.1419 | 0.0090 | - | 0.0090 | 0.0091 | 0.0091 | 0.0090 | 0.0090 | 0.0092 | 0.0092 | 0.0091 | 0.0091 | 0.0091 | 0.0091 | 0.0091 | 0.0091 | 0.0091 | 0.0091 | 0.0091 | 0.0091 | 0.0092 |
| **AUS. MTB** | 0.0088 | 0.0088 | 0.0134 | 0.0088 | - | 0.0088 | 0.0089 | 0.1423 | 0.0089 | 0.0088 | 0.0090 | 0.0089 | 0.0089 | 0.0089 | 0.0089 | 0.0089 | 0.0089 | 0.0090 | 0.0089 | 0.0089 | 0.0088 | 0.0088 |
| **AUS. SLB** | 0.0117 | 0.0118 | 0.0120 | 0.0119 | 0.0120 | - | 0.0587 | 0.0358 | 0.0118 | 0.0119 | 0.0120 | 0.0119 | 0.0117 | 0.0118 | 0.0120 | 0.0121 | 0.0118 | 0.0120 | 0.0120 | 0.0119 | 0.0118 | 0.0120 |
| **AUS. TBC** | 0.0063 | 0.0064 | 0.0064 | 0.0062 | 0.0063 | 0.0063 | - | 0.1363 | 0.0063 | 0.0064 | 0.0063 | 0.0064 | 0.0063 | 0.0063 | 0.0062 | 0.0062 | 0.0063 | 0.0063 | 0.0063 | 0.0063 | 0.0063 | 0.0063 |
| **AUS. TDR** | 0.0067 | 0.0067 | 0.0072 | 0.0068 | 0.0068 | 0.0068 | 0.0069 | - | 0.0067 | 0.0067 | 0.0068 | 0.0070 | 0.0067 | 0.0068 | 0.0067 | 0.0068 | 0.0069 | 0.0068 | 0.0067 | 0.0069 | 0.0069 | 0.0068 |
| **AUS. TRI** | 0.0093 | 0.0092 | 0.1243 | 0.0093 | 0.0092 | 0.0092 | 0.0093 | 0.0235 | - | 0.0094 | 0.0092 | 0.0093 | 0.0093 | 0.0093 | 0.0093 | 0.0093 | 0.0093 | 0.0091 | 0.0093 | 0.0092 | 0.0092 | 0.0093 |
| **CHN. DNZ** | 0.0098 | 0.0097 | 0.0099 | 0.0099 | 0.0097 | 0.0099 | 0.0097 | 0.0098 | 0.0098 | - | 0.1268 | 0.0098 | 0.0098 | 0.0098 | 0.0098 | 0.0098 | 0.0097 | 0.0098 | 0.0098 | 0.0102 | 0.0097 | 0.0098 |
| **CHN. DZH** | 0.0075 | 0.0074 | 0.0074 | 0.0075 | 0.0074 | 0.0073 | 0.0075 | 0.0073 | 0.0074 | 0.0075 | - | 0.0075 | 0.0075 | 0.0073 | 0.0074 | 0.0074 | 0.0075 | 0.0074 | 0.0075 | 0.0075 | 0.0075 | 0.0074 |
| **FJ. SVP** | 0.0093 | 0.0093 | 0.0094 | 0.0094 | 0.0094 | 0.0094 | 0.0093 | 0.0097 | 0.0095 | 0.0094 | 0.0095 | - | 0.0094 | 0.0094 | 0.0094 | 0.0092 | 0.0094 | 0.0094 | 0.0094 | 0.0096 | 0.0092 | 0.0093 |
| **IDN. NSL** | 0.0085 | 0.0084 | 0.0085 | 0.0084 | 0.0086 | 0.0084 | 0.0085 | 0.0084 | 0.0084 | 0.0084 | 0.0085 | 0.0085 | - | 0.0085 | 0.0084 | 0.0085 | 0.0085 | 0.0087 | 0.0086 | 0.0276 | 0.0084 | 0.0085 |
| **JPN. IRT** | 0.0069 | 0.0069 | 0.0069 | 0.0070 | 0.0069 | 0.0070 | 0.0069 | 0.0068 | 0.0070 | 0.0069 | 0.0070 | 0.0068 | 0.0070 | - | 0.0070 | 0.0069 | 0.0069 | 0.0069 | 0.0069 | 0.0069 | 0.0069 | 0.0069 |
| **KRB. BTR** | 0.0083 | 0.0083 | 0.0083 | 0.0084 | 0.0083 | 0.0083 | 0.0083 | 0.0084 | 0.0083 | 0.0082 | 0.0084 | 0.0083 | 0.0083 | 0.0082 | - | 0.0086 | 0.0083 | 0.0083 | 0.0084 | 0.0083 | 0.0083 | 0.0084 |
| **KRB. TRW** | 0.0078 | 0.0079 | 0.0078 | 0.0079 | 0.0079 | 0.0079 | 0.0078 | 0.0079 | 0.0078 | 0.0079 | 0.0078 | 0.0079 | 0.0079 | 0.0079 | 0.0104 | - | 0.0080 | 0.0078 | 0.0079 | 0.0079 | 0.0079 | 0.0079 |
| **MCN. CHK** | 0.0078 | 0.0078 | 0.0078 | 0.0078 | 0.0078 | 0.0079 | 0.0149 | 0.0078 | 0.0078 | 0.0078 | 0.0078 | 0.0077 | 0.0078 | 0.0152 | 0.0109 | 0.0090 | - | 0.0080 | 0.0078 | 0.0085 | 0.0078 | 0.0078 |
| **MCN. KSR** | 0.0098 | 0.0099 | 0.0097 | 0.0098 | 0.0099 | 0.0098 | 0.0137 | 0.0098 | 0.0098 | 0.0098 | 0.0098 | 0.0097 | 0.0098 | 0.0098 | 0.1218 | 0.0099 | 0.0109 | - | 0.0097 | 0.0098 | 0.0098 | 0.0098 |
| **MCN. YAP** | 0.0076 | 0.0078 | 0.0076 | 0.0075 | 0.0076 | 0.0076 | 0.0076 | 0.0077 | 0.0077 | 0.0076 | 0.0077 | 0.0076 | 0.0077 | 0.0076 | 0.0076 | 0.0076 | 0.0076 | 0.0077 | - | 0.0077 | 0.0077 | 0.0076 |
| **MLS. TKP** | 0.0090 | 0.0084 | 0.0084 | 0.0091 | 0.0096 | 0.0092 | 0.0100 | 0.0084 | 0.0092 | 0.0091 | 0.0095 | 0.0085 | 0.0121 | 0.0092 | 0.0101 | 0.0086 | 0.0084 | 0.0104 | 0.0085 | - | 0.0091 | 0.0085 |
| **PHP. PNL** | 0.0084 | 0.0083 | 0.0084 | 0.0084 | 0.0083 | 0.0084 | 0.0082 | 0.0085 | 0.0083 | 0.0084 | 0.0475 | 0.0083 | 0.0466 | 0.0697 | 0.0084 | 0.0083 | 0.0083 | 0.0084 | 0.0083 | 0.0189 | - | 0.0084 |
| **USA. GM** | 0.0083 | 0.0082 | 0.0084 | 0.0083 | 0.0083 | 0.0084 | 0.0083 | 0.0082 | 0.0081 | 0.0083 | 0.0083 | 0.0084 | 0.0083 | 0.0082 | 0.0083 | 0.0083 | 0.0084 | 0.0083 | 0.0085 | 0.0083 | 0.0083 | - |


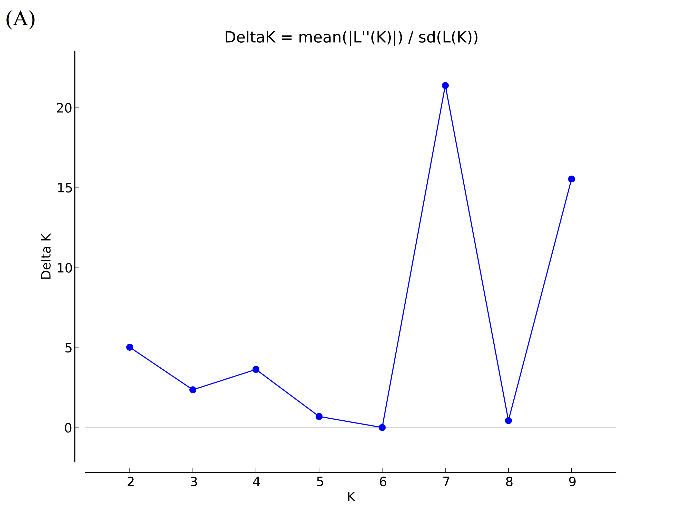

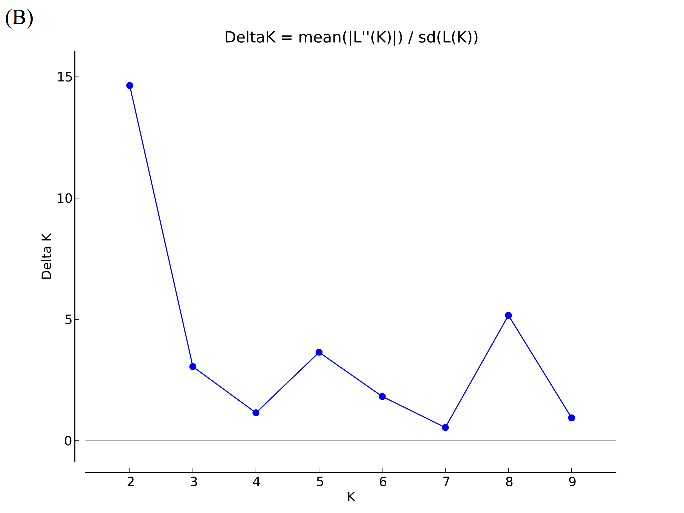

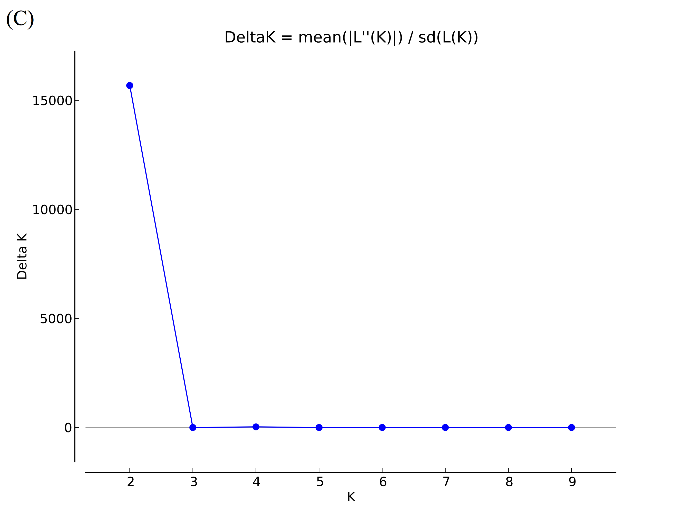


**Supplementary Figure 1.** Plots of delta K across different Ks for *R. apiculata* (A), *R. mucronata* (B), and *R. stylosa* (C). The optimal Ks determined by Evanno’s method for populations of *R. apiculata*, *R. mucronata*, and *R. stylosa* are 7, 2, and 2, respectively.


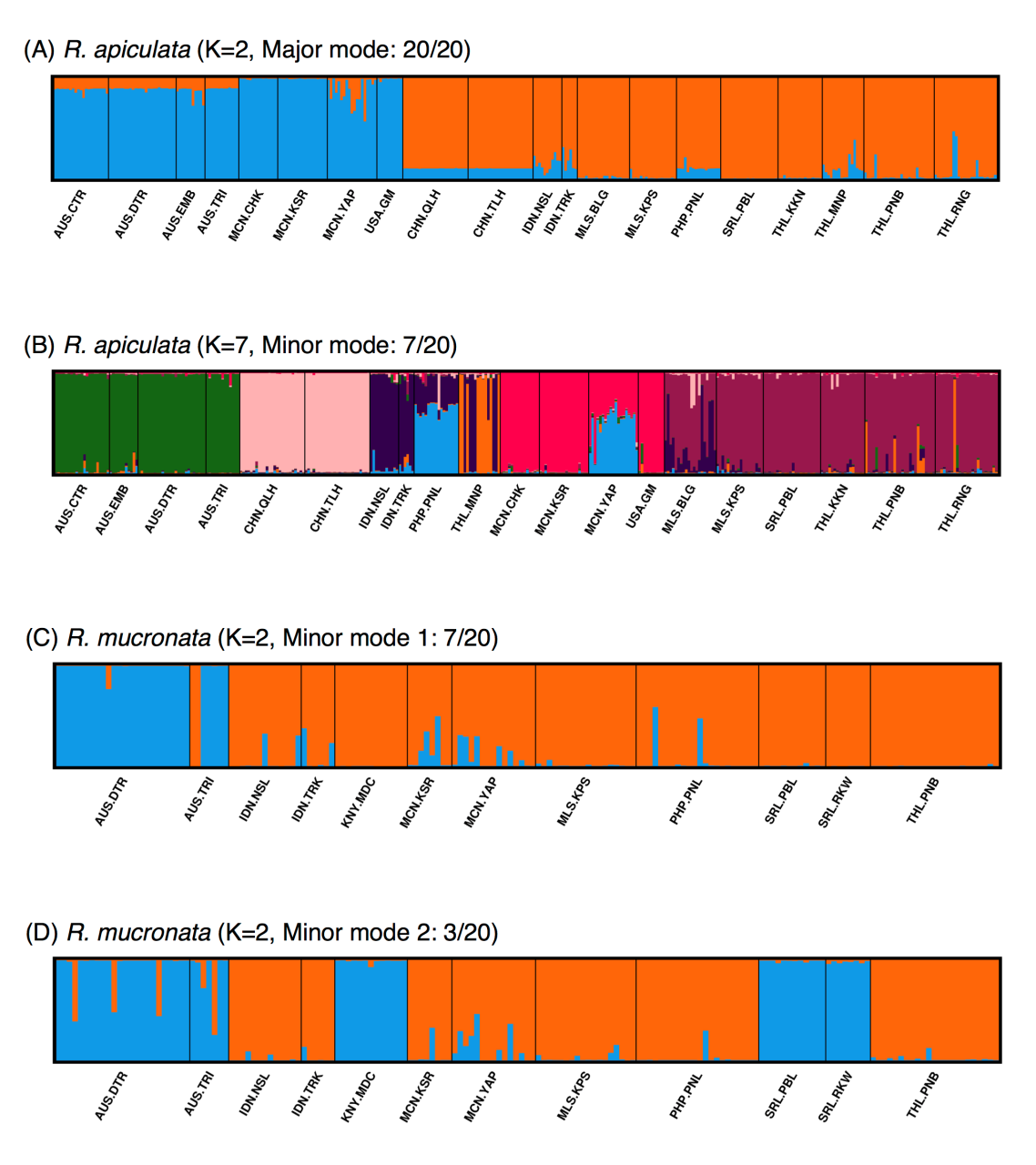


**Supplementary Figure 2.** STRUCTURE bar plot showing the assignment of individuals of *R. apiculata* when K = 2 (A), and the minor mode of assignment of individuals of *R. apiculata* when K = 7 (B). C and D are two minor modes of assignment of individuals of *R. mucronata* when K = 2.


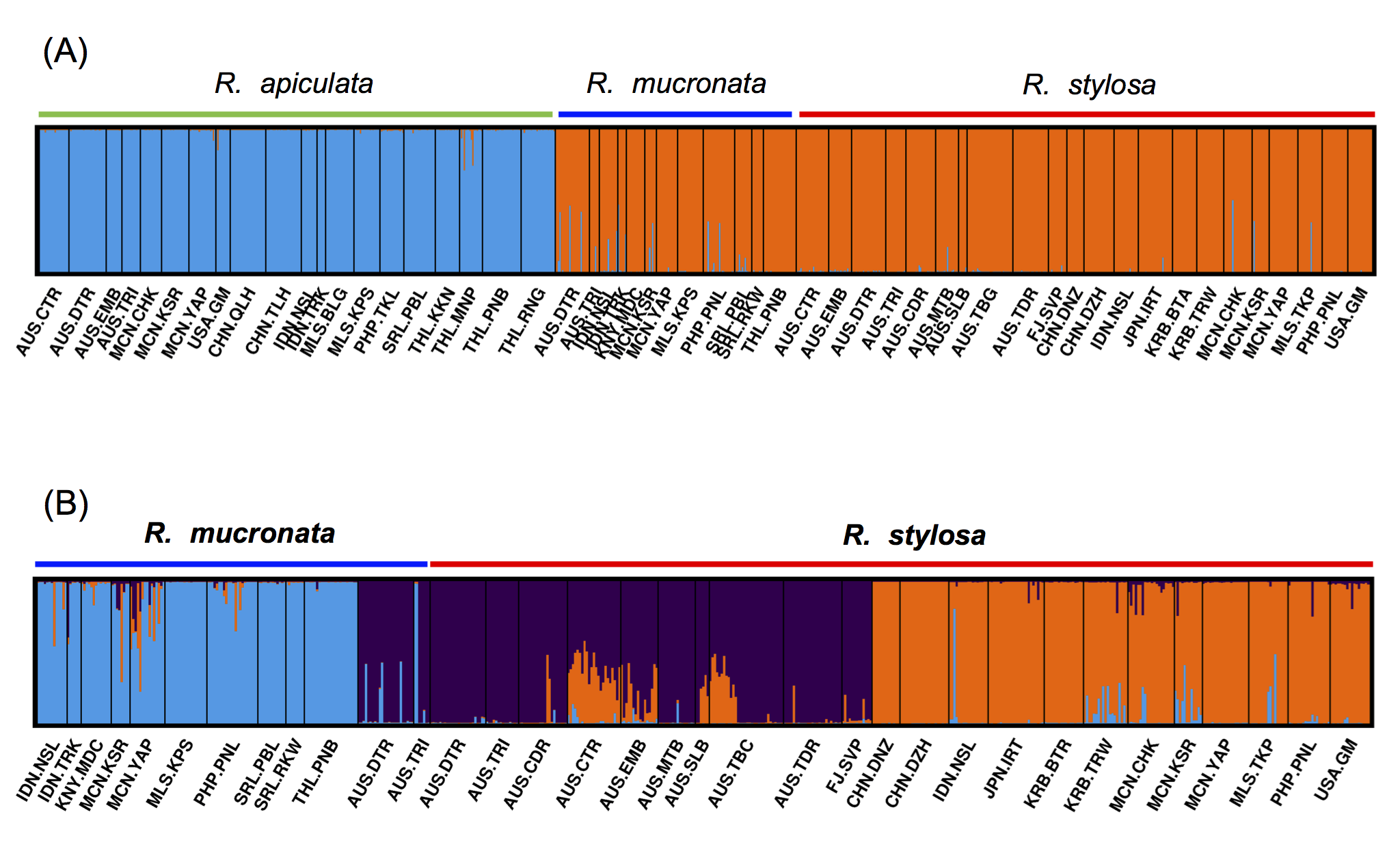


**Supplementary Figure 3.** STRUCTURE bar plot showing assignment of individuals for all three species combined (best K = 2; A), and for *R. mucronata* and *R. stylosa* combined (best K = 3; B).


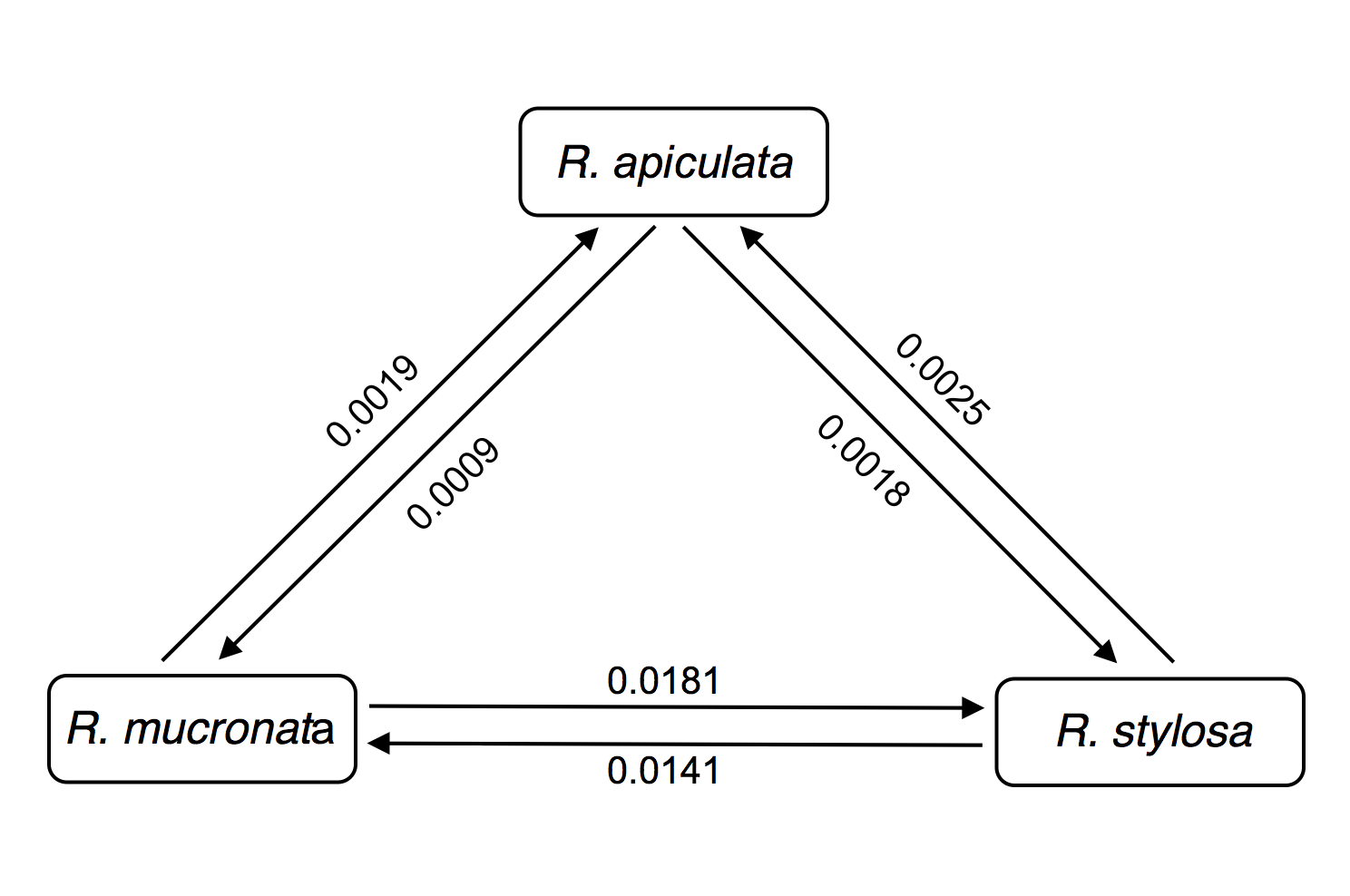


**Supplementary Figure 4.** Estimated migration rates (Nm, number of migrates per generation) between pair-wise taxa using BAYESASS.
